# Plasticity of primary and secondary growth dynamics in *Eucalyptus* hybrids: a quantitative genetics and QTL mapping perspective

**DOI:** 10.1186/1471-2229-13-120

**Published:** 2013-08-26

**Authors:** Jérôme Bartholomé, Frédéric Salmon, Philippe Vigneron, Jean-Marc Bouvet, Christophe Plomion, Jean-Marc Gion

**Affiliations:** 1 CIRAD, UMR AGAP, F-33612 Cestas, France; 2 INRA, UMR BIOGECO, F-33612 Cestas, France; 3 CIRAD, UMR AGAP, F-34398 Montpellier, France; 4 CIRAD, UMR AGAP, Pointe Noire, Congo; 5 CRDPI, BP 1291 Pointe Noire, Rep. of Congo

**Keywords:** Growth, Growth modelling, Plasticity, QTL x E interaction, Heritability, Eucalyptus

## Abstract

**Background:**

The genetic basis of growth traits has been widely studied in forest trees. Quantitative trait locus (QTL) studies have highlighted the presence of both stable and unstable genomic regions accounting for biomass production with respect to tree age and genetic background, but results remain scarce regarding the interplay between QTLs and the environment. In this study, our main objective was to dissect the genetic architecture of the growth trajectory with emphasis on genotype x environment interaction by measuring primary and secondary growth covering intervals connected with environmental variations.

**Results:**

Three different trials with the same family of *Eucalyptus urophylla* x *E. grandis* hybrids (with different genotypes) were planted in the Republic of Congo, corresponding to two QTL mapping experiments and one clonal test. Height and radial growths were monitored at regular intervals from the seedling stage to five years old. The correlation between growth increments and an aridity index revealed that growth before two years old (r = 0.5; 0.69) was more responsive to changes in water availability than late growth (r = 0.39; 0.42) for both height and circumference. We found a regular increase in heritability with time for cumulative growth for both height [0.06 - 0.33] and circumference [0.06 - 0.38]. Heritabilities for incremental growth were more heterogeneous over time even if ranges of variation were similar (height [0-0.31]; circumference [0.19 to 0.48]). Within the trials, QTL analysis revealed collocations between primary and secondary growth QTLs as well as between early growth increments and final growth QTLs. Between trials, few common QTLs were detected highlighting a strong environmental effect on the genetic architecture of growth, validated by significant QTL x E interactions.

**Conclusion:**

These results suggest that early growth responses to water availability determine the genetic architecture of total growth at the mature stage and highlight the importance of considering growth as a composite trait (such as yields for annual plants) for a better understanding of its genetic bases.

## Background

Production of wood biomass is a mandatory trait in any forest tree breeding program regardless of the final use be it pulp and paper, energy, construction or engineered wood products. Understanding the contribution of genetic and environmental factors as well as their interaction in tree growth and adaptation is a prerequisite for accelerating tree domestication to meet the increasing demand for wood [1]. In this context, fast growing trees such as *Eucalyptus* will play a major role, not only as wood supply, but also as a model system to decipher the determinism of growth. Indeed, eucalypts are cultivated worldwide on more than twenty million hectares and are the most planted hardwoods in the world [2]. This genus comprises about 700 species [3] distributed naturally over a wide range of pedoclimatic conditions. Within that diversity, a few species or inter-specific hybrids combining the adaptive capacities and fast growth rates of the parental species (e.g. *E. urophylla* x *E. grandis*[4,5]), are widely used in planted forest.

Wood biomass formation is a dynamic process resulting from a wide array of physiological processes [6] some of which (e.g. photosynthesis) are dependent on environmental variations. It is also a product of height and circumference growth, involving the activity of the shoot apical meristem [7] and the vascular cambium [8]. The dynamics of both primary and secondary growth have been extensively described and modelled up to the rotation age in many forest tree species [9-12] with the main objectives of predicting biomass production and optimizing sylvicultural practices. The first models corresponded to yield models and with the improved understanding of plant/tree physiological functioning and its interplay with climatic factors, process or mechanistic models were subsequently developed taking into account carbon allocation, nutrient and water availability, and climate effects [13,14]. These models were often developed for a particular species [15,16] without explicitly taking into account the genetic diversity within species, while a genetic effect of the response to environmental variation has been described in *Eucalyptus* on different scales of growth analysis (day, month, year) [17,18].

The genetic determinism of tree growth and its trend over-time have been fairly well studied in most forest tree species of commercial interest [19-24]. As regards time trends, the Franklin model [25], suggesting three phases in variance components for growth traits (juvenile, mature and adult phases), was tested in different species [26-28]. In short rotation species with rapid initial growth such as in *Eucalyptus*, a more stable trend was usually observed up to the rotation age [23]. Similarly, the ratio between dominance and additive variances has been analysed over time, with a major share of additive variance in *Pinus taeda*[29] and more balanced in *Eucalyptus*[23]. Age-age genetic correlations were found to be generally high between successive measurements and tended to decrease when the time lag increased i.e. between juvenile and mature stages [27,30,31]. Overall, these studies reveal low to moderate heritability for growth traits, indicating a strong environmental effect on the determinism of growth. More recent studies have tried to link genetic of growth response to environmental variation [23,32,33]. In *Eucalyptus globulus*, Costa e Silva *et al*. [34] reported a genetic effect on radial growth in response to temperature, rainfall and solar radiation. A similar result was presented by a more recent study showing a genetic effect for suceptibility to drought damage in *E. globulus*[35]. Nevertheless, there is still a need to improve our understanding of genetic and environmental determinants of growth, through measurements covering intervals better connected with environmental variations.

An analysis of the genetic architecture of growth traits (i.e. the number, map location and effect of quantitative trait loci, QTL) can also provide a better understanding of the genetic basis of growth. Thus, over the past two decades, many studies have been undertaken to detect QTLs controlling part of the phenotypic variation of wood biomass in forest trees such as pines [36], poplar [37] and eucalyptus [38]. In *Eucalyptus*, several genomic regions have been detected harbouring QTLs with low to moderate effects on the phenotypic variation of growth traits [39-44]. These studies, although carried out with a relatively small number of growth measurement time points revealed QTL instability over time [40,41]. The functional mapping approach taking into account the growth trajectory in QTL analysis [45] showed similar results with early and late QTLs in poplar [46]. However, rather few studies connecting the genetic architecture of growth traits and different environmental conditions have been carried out [47-50]. In *Eucalyptus* Freeman *et al*. [51,52] as well as Teixeira *et al*. [53], highlighted major QTL x Environment interactions. All these studies conducted on an inter-annual scale are consistent with the idea that growth is an integrative trait involving a large number of ecophysiological [6] and molecular [54] processes on which environmental conditions can act to determine the final genetic architecture of growth. Therefore, understanding the interplay between early growth and abiotic factors (e.g. alternation between dry and wet seasons in the tropics) and how environmental variations impact the genetics of growth is a key issue, especially as part of *Eucalyptus* breeding programmes.

In this context, the objective of this study was to dissect the genetic basis of the growth trajectory and its interplay with seasonal variation in water availability. To that end, one interspecific cross of *Eucalyptus urophylla* x *E. grandis* was planted in three different field trials in the Republic of Congo where contrasting environmental conditions in term of water availability (rainy vs. dry seasons), were suitable for observing the effect of drought on growth. We used two complementary types of growth trait: integrative traits (cumulative growth, growth curve parameters) and responsive traits (sub-annual growth increments) in combination with climatic data to characterize growth response to changes in water availability and reveal its genetic component. This comprehensive dissection of growth enabled us to characterize the genetic determinism of primary (height) and secondary (circumference) growth using conventional quantitative genetics and QTL detection approaches.

## Methods

### Plant material and field experiments

A full-sib family generated from a single controlled cross between *Eucalyptus urophylla* (accession 14.144) x *E. grandis* (accession 9.21) was used in this study in three environmental settings corresponding to three field experiments. The first two trials were planted using a single tree plot design with seedlings: in April 1993 with 201 genotypes (referred to as P93) and already used in previous QTL studies [40,55,56] and in April 1997 (P97) with 190 genotypes (different from P93). The third trial was planted in November 1998 (P98) and corresponded to a clonal test with 60 genotypes in common with P93 replicated by rooted-cuttings with 10 copies per genotype.

The three trials were established in the Republic of Congo, in the Pointe-Noire region (4.8° S, 12° E). P93 and P97 were planted at a density of 667 trees/ha, whereas P98 was planted at 800 trees/ha. To reduce border effects a triple border row was planted around all the plantations.

DNA extractions for the full-sib progenies were carried out using dry leaves according to Doyle and Doyle [57].

### Environmental characterization

The trials were planted on Ferralic Arenosols (FAO classification) at the Kissoko forestry station (Pointe-Noire, Republic of Congo). This soil is considered to be poor due to a low water retention, a small cationic exchange capacity and a low organic matter content [58]. The climate in the Pointe-Noire region corresponds to a tropical savanna climate [59] characterized by a dry season from May to October with annual rainfall of about 1,200 mm. The high atmospheric humidity (85% on average) and high temperature (25°C) display slight seasonal variations, at 2% and 3°C, respectively. To compare the climate between trials and especially the length of dry period during the dry season, we used the De Martonne aridity index (I_DM_). I_DM_ was calculated monthly with the following formula [60]:

$$I_{\mathrm{DM}}=12\;p/(t+10)$$

where p is the total monthly rainfall and t the monthly mean temperature. According to De Martonne [60], a threshold of I_DM_ =15 was chosen as an upper limit to define the dry season. Climatic data (temperature and rainfall) were recorded at Pointe-Noire about 25 km from the field trials.

### Phenotypic traits

#### Growth Measurements

Tree growth was assessed by measuring both cumulative height and circumference at 1.3 m for each tree, at different ages. P93 was measured at 14, 26, 39, 51 and 59 months old. P97 was measured more frequently over time at 7, 8, 9, 10, 11, 12, 13, 15, 18, 21, 24, 27, 30, 34, 44, 52 and 62 months old. P98 was measured at 6, 12, 15, 25, 28, 33, 36, 42, 48, and 60 months old. The net growth increments between two time points were calculated using the following formulas:

$$\text{Trait}\;n_{1-}n_{2}=\text{Trait}\;n_{2}-\text{Trait}\;n_{1}$$

Where Trait n is the cumulative height or circumference at “n” months old.

#### Growth modelling

We used the monomolecular growth equation to model height and circumference growth in trials P93, P97 and P98. The monomolecular growth equation is a particular case of the von Bertalanffy-Richards growth model [61,62] which has been widely applied and compared for tree growth modelling [63-67]. For fast growing trees like eucalypts, the monomolecular model is well adapted because of its concave shape with no inflection point [68-70]. The equation is expressed in the following form:

$$l\left(t\right)=\mathrm{Asym}\;\left[1-e^{-e}{}^{{}^{\mathrm{lrc}}\left(t‒c0\right)}\right]$$

Where *l(t)* is the cumulative growth at time t; Asym is the maximum growth (the asymptote); lrc is the logarithm of the growth constant and c0 is the theoretical age for which *l(t)* is zero. Growth modelling was performed with *R* software (Version 2.15.2; http://cran.r-project.org/) using the *R* package: *nlme*[71,72]. Trees with a high proportion of missing data were removed, based on the duration and the phase (exponential growth phase or asymptotic phase) at which missing data occurred. Selected trees presented on average 0.3, 2.2 and 0.5% of growth curve that is missing for P93, P97 and P98 respectively. For each selected genotype, a nonlinear model (monomolecular) was fitted to obtain the above-mentioned growth curve parameters (GCP) for both height and circumference. The model fitted the data well as illustrated for circumference in Additional file 1. Moreover the pseudo R-squared calculated for all adjusted models were higher than 0.9. Predicted values were also calculated for P97 for the same time points as in P93 (14, 26, 39, 51 and 59 months old).

#### Broad-sense heritability of growth

Broad-sense heritabilities were estimated using trial P98 which corresponded to a clonal test with 10 replicates of 60 full-sibs. Analyses were conducted using Markov Chain Monte Carlo methods implemented in the R *MCMCglmm* package [73]. The following mixed effects model was used for each trait:

$$y=µ+{\mathrm{Row}}_{i}+{\mathrm{Col}}_{j}+G_{k}+\epsilon$$

where y is the individual tree measurement, μ is the general mean, Row_i_ is the fixed effect of the row, Col_j_ is the fixed effect of the column, G_*k*_ is the random effect of the genotype $\sim N\left(O,\sigma_{G}^{2}\right)$ and ϵ is the residual random effect $\sim N\left(O,\sigma_{\epsilon }^{2}\right)$. The intersection between individual tree coordinates in the trial (row and column) represented the spatial position of each tree. Broad-sense heritability for the full-sib family (H^2^) was estimated for each trait using the following formula:

$$H^{2}=\sigma_{G}^{2}/\left(\sigma_{G}^{2}+\sigma_{\epsilon }^{2}\right)$$

Where $\sigma_{G}^{2}$ is the genetic variance and $\left(\sigma_{G}^{2}+\sigma_{\epsilon }^{2}\right)$ is the phenotypic variance [74].

### Molecular markers and linkage mapping

For each parental genotype (*E. urophylla* and *E. grandis*) and each trial (P93 and P97), a genetic map was constructed according to a two-way pseudo-test cross mapping strategy [75]. For P93, the two genetic linkage maps were constructed using RAPD SSR, EST, and STS markers genotyped on 201 individuals [56]. For P97, only RAPD markers selected on the basis of their map position in the P93 linkage maps were used for genetic mapping with 190 individuals. The set of common polymorphic markers genotyped in both P93 and P97, enabled the unambiguous identification of homologous linkage groups (LGs) between the two parental maps of the same species. In addition, the codominant markers used in P93, enabled the identification of orthologous regions between *E. urophylla* and *E. grandis*[56].

JoinMap® version 4.1 [76,77] was used to perform the linkage analysis. Chi-squared tests were performed to test whether markers followed the expected Mendelian segregation ratios. Distorted markers (p <0.01) were excluded from linkage analysis. Markers with more than 40% of missing data were also excluded. For the four maps, markers were grouped into LGs at the independence logarithm-of-the-odds (LOD) score ≥ 4.0. Then, markers within linkage groups were ordered using the Monte Carlo maximum likelihood mapping algorithm [78,79]. Finally, mapping quality tools were used to classify markers leading to a poorer fit as accessory markers, located near the closest framework markers. Accessory markers with significant deviation from expected Mendelian segregation (p < 0.01) were indicated by * on the genetic maps. Genetic distances in centiMorgan (cM) were calculated with the Kosambi mapping function [80]. The LG nomenclature corresponds to that defined by Brondani *et al*. [81].

### QTL analysis

#### QTL mapping

Quantitative trait locus (QTL) analyses were performed with *R* software using the *qtl* package [82]. We carried out simple and composite interval mapping using the multiple imputation method of Sen and Churchill [83] with a step interval of 1 cM, a genotyping error rate of 0.001 and 512 imputations per genotypes. It has been shown that this imputation method performs better with missing genotypic data [83]. The whole-genome significance thresholds for all traits were determined with 10,000 permutations [84]. QTLs that met or exceeded 95^th^ and 90^th^ percentiles were assessed to be significant or suggestive, respectively. Confidence intervals for each QTL position were obtained using Bayes credible intervals (BCI). Genetic maps and QTL positions were drawn using MapChart [85].

#### Detection of QTL x environment interaction

For each parent, we were able to test the QTL x environment interaction (QTL x E) using markers common to both trials (P93 and P97), and for growth traits at five different ages (14, 26, 31, 51, 59 months old) based on measurements for P93 and predicted values at the same ages for P97. GCP were also used for both trials. To test the QTL x E, an analysis of variance (*lm* and *anova* function, R software) was carried out according to the following model:

y=µ+Trial+Marker+Trial×Marker+ϵ

where y is the individual tree measurement, μ is the general mean, Trail is the fixed effect of the trial (P93 or P97), Marker is the fixed effect of the closest marker associated with a QTL detected by CIM, Trail × Marker is the interaction between the two fixed effects and ϵ is the residual random effect $\sim N\left(O,\sigma_{\epsilon }^{2}\right)$.

## Results

### Climatic conditions

**Figure 1:**
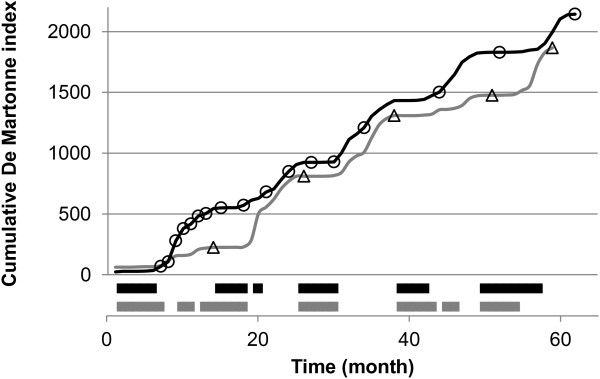
**Comparison of seasonal changes in water availability between P93 and P97, using the I**_**DM**_**.** Measurement points are represented by triangles for P93 (grey) and circles for P97 (black). The coloured bars (x-axis) represent the dry periods (I_DM_ < 15) for each trial with the same colour code.

The De Martonne index (I_DM_) was calculated monthly during the growth period of P93 and P97 (Figure 1). I_DM_ mainly reflected seasonal changes in water availability because the mean temperature remained very stable across seasons with only 3°C of amplitude. The two curves exhibited different patterns at the beginning of the plantation (between 8 and 20 months), then evolved in parallel before separating again after 45 months. The timing and the intensity of the dry periods (defined as I_DM_ < 15) were different between the two trials. Indeed, P93 exhibited longer dry periods than P97 (except for the third and the last dry seasons), amounting to four supplementary months of drought over the study period. Using the dry periods defined by I_DM_, specific growth increments were determined for P97 only, because measurement points were lacking for P93.

### Trend in phenotypic variation and correlation

**Figure 2:**
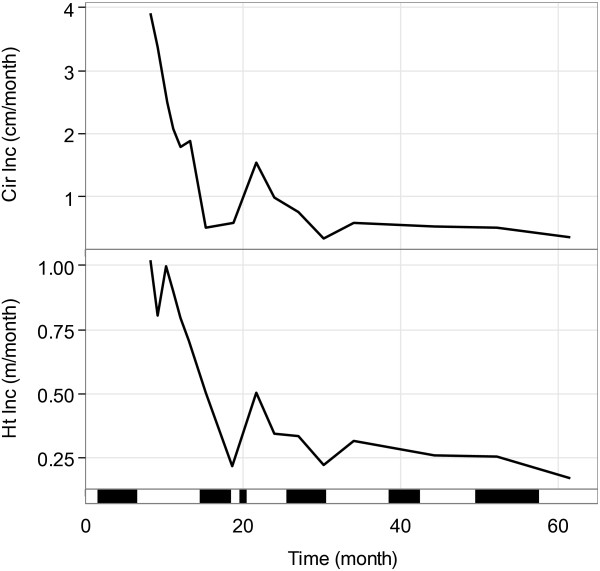
**Mean trajectories for height (Ht) and circumference (Cir) increments for the P97 trial.** The thick black bars represent dry periods (I_DM_ < 15).

The mean trajectories for cumulative growth, height (Ht) and circumference (Cir), were slightly different between the three trials (Additional file 2). Mean Cir values were significantly smaller in P98 (p < 0.01) whereas final Ht values at 60 months old were higher (p < 0.01), probably due to a higher tree density in P98. These differences were reduced between P93 and P97, two trials planted at the same period (April) and with the same tree density. The mean trajectory of growth increments, estimated from P97 (Ht_Inc and Cir_Inc), showed a global decrease over the 60 months after planting, with two periods of lower growth rates that occurred during the second and third dry periods (Figure 2). These two periods flanked a growth rate peak at 21 months that occurred during the second rainy season.

The pattern of phenotypic variations for cumulative growth (Ht, Cir) was very similar between trials. Indeed the variance increased gradually over time and the coefficient of phenotypic variation (CVp) for Ht remained rather stable ([0.07-0.09], [0.13-0.19] and [0.12-0.24] for P93, P97 and P98, respectively, Additional file 3). The same tendency was observed for Cir. The CVp of growth increments for P97 and P98 were more variable compared to cumulative growth, as exemplified by CVp values for Ht_Inc ranging from 0.18 to 0.58 for P97 and from 0.12 to 0.82 for P98. A similar range of values was observed for Cir_Inc. Given the large number of measurements in P97, we were able to characterize the evolution of CVp over time for Ht_Inc and Cir_Inc. Two growth rate peaks CVp were observed during the second and the third dry periods (Additional file 4), corresponding to a lower growth increment on average. These results suggest that growth rate differences between genotypes were enhanced during these two dry periods.

**Figure 3:**
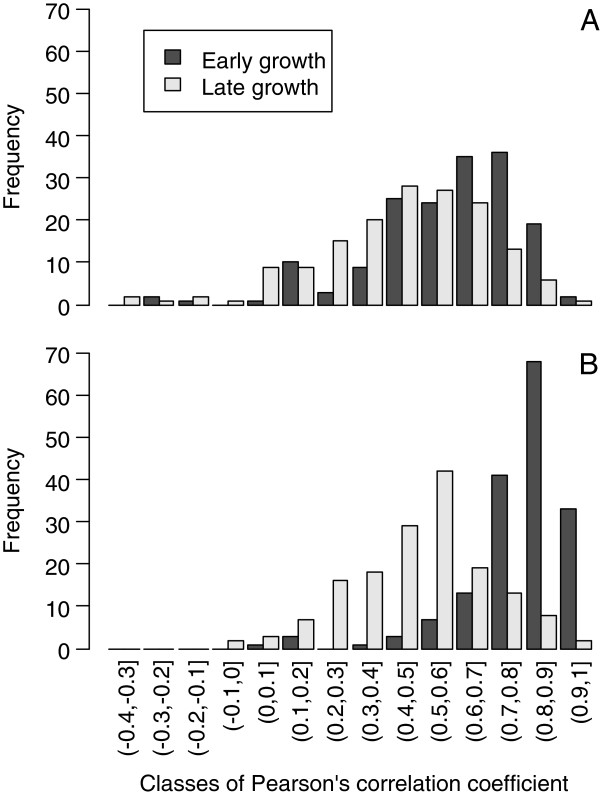
**Distribution of Pearson’s correlation coefficients between growth increments and I**_**DM**_**.** Pearson’s coefficients of correlation were calculated for early (< 24 months) and late (>24 months) growth for height **(A)** and circumference **(B)**.

This hypothesis was supported by the analysis of Pearson correlations between height or radial increments and I_DM_ calculated at both trial and tree levels using data from P97 (Table 1). At trial level (i.e. considering all data points for all trees), positive correlations were found for both early (<24 months) and late (>24 months) growth, indicating a strong effect of rainfall on growth. For both levels, early growth was found to be more responsive (higher correlations) than late growth, although fewer data were available at the later stage, which could have affected the estimation of correlation coefficients. The tree level reflected the phenotypic plasticity of each genotype in response to water availability (1 = highly responsive, non-significant = non responsive). At tree level, correlations between I_DM_ and circumference increments were higher than those with height increments (Figure 3). These differences were particularly apparent during early growth with 95% of trees having a coefficient of correlation over 0.5, including 81% of trees with a significant correlation (p-value < 0.05). This suggests that secondary growth was more prone to changes in water availability than primary growth, especially at the juvenile stage.

**Table 1:** **Pearson’s coefficients of correlation (r) between growth increments and I**_**DM**_

		**Trial level**	**Tree level**	
		**Pearson’s r (p-value)**	**Mean of Pearson’s r [range]**	**% of significant correlation**
Height increment	Early growth	0.5 (2.2e-16)	0.58 [-0.26, 0.96]	32.3%
	Late growth	0.39 (2.2e-16)	0.44 [-0.36, 0.9]	5.7%
Circumference increment	Early growth	0.69 (2.2e-16)	0.79 [0.02, 0.98]	81.1%
	Late growth	0.42 (2.2e-16)	0.49 [-0.08, 0.92]	7.5%

Pearson’s coefficients of correlation were calculated for early (< 24 months) and late (>24 months) growth at trial and tree levels.

**Figure 4:**
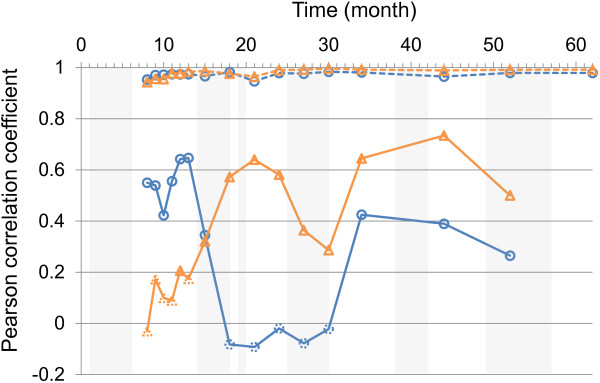
**Evolution of Pearson’ coefficients of correlations for the P97 trial.** Correlations were calculated between cumulative growth measurements at age n and n + 1 (dashed line) and between growth increments between age n-1_n and n_n + 1 (solid line). Height is in blue with circles, circumference is in orange with triangles. Non-significant correlation values with p-value > 0.05 are indicated with symbols in a dotted line. Vertical grey bars indicate the dry periods.

The analysis of phenotypic correlations between cumulative growth (Ht and Cir) revealed strong correlations (>0.78) over time in each trial. As expected, the age–age correlations for both cumulative growth traits (Ht and Cir) were highly positive and stable over time, and comparable between the three trials (Additional file 5; A,B,C). For Ht and Cir, the correlations between current age and final growth increased dramatically over the two first years to reach at least 0.8 at two years old (Additional file 6). High correlations were also found between Asym and the final growth. More interestingly, Pearson correlation coefficients for growth increments were variable over time. Moreover, correlations for Ht_Inc and Cir_Inc displayed roughly opposite patterns during early growth (until the third dry period). As illustrated for P97 in Figure 4, correlations for Ht_Inc dropped from high and significant levels (maximum of 0.64 at 13 months old) to non-significant levels revealing contrasting behaviour between genotypes in their response to the environment between the beginning of the second dry period and the 34^th^ month. Conversely, correlations for Cir_Inc became significant during the same period revealing a similar response between genotypes.

### Trend in the genetic control of growth traits

**Figure 5:**
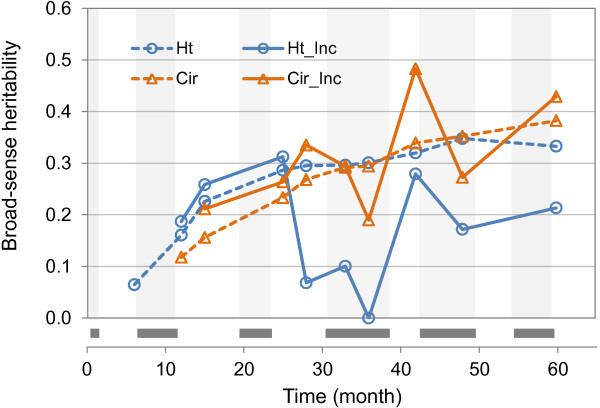
**Time trend in broad sense heritabilities (H**^**2**^**) for height and circumference for the P98 trial.** Cumulative growth is in a dashed line and incremental growth is in a solid line. Height (Ht) is in blue with circles, circumference (Cir) is in orange with triangles. Vertical grey bars indicate the dry periods.

Broad-sense heritabilities (H^2^) were estimated for cumulative growth traits, growth increment, and the GCP based on the data from P98. For all traits, H^2^ values were small to medium. Although standard errors associated with H^2^ were high (± 0.1 on average), we observed a regular increase in heritability with time for cumulative growth: Ht [0.06-0.33], Cir [0.12 to 0.38] (Figure 5, Additional file 7). The heritabilities for the Asym parameter (Asym_h: 0.35 and Asym_c: 0.44) were only slightly higher than those obtained for final Ht and Cir, which is consistent with the strong correlation between these pairs of traits. H^2^ for growth increments were more heterogeneous than H^2^ for cumulative traits (Figure 5, Additional file 7), even though the variation ranges were similar (Ht_Inc [0-0.31]; Cir_Inc [0.19 to 0.48]). Given the standard error around H^2^, only differences between cumulative and incremental growth for Ht (at ages 28, 33, 36 and 48) were found to be significant between the two types of trait, while no significant differences for Cir were observed. H^2^ for Ht_Inc and Cir_Inc had nearly an opposite pattern from 25 to 35 months, then their patterns became more similar. The lowest heritabilities for Ht and Cir clearly corresponded to the coefficient of variation peak occurring during the third dry season (Additional file 4) and suggest a greater environmental effect at that period.

### Linkage mapping

The linkage analysis was performed for the P93 and P97 trials separately. Table 2 summarizes the characteristics of each genetic map. For P93, 57 co-dominant markers mapped by Gion *et al*. (2011) enabled the identification of orthologous regions between *E. urophylla* and *E. grandis* parents. These regions covered a total of 989 cM for *E. urophylla* and 847 cM *E. grandis*, amounting respectively to 66% and 67% of the total map length. The macro-colinearity of common markers between parental maps was well conserved except for 6 small inversions in LGs 2, 5 and 7 (Additional file 8). For P97, the two parental maps were constructed in this study, using a total of 148 RAPD markers for *E. urophylla* and 130 RAPDs for *E. grandis*. A total of 106 and 86 framework markers were positioned in 13 (chromosome 2 and 11 were split into two sub-groups) and 11 linkage groups (LGs) for *E. urophylla* and *E. grandis* parents respectively. The two maps provided a resolution of one marker every 14.5 cM (*E. urophylla*) and 15.3 cM (*E. grandis*) for a total map length of 1,179 cM (*E. urophylla*) and 1,021 cM (*E. grandis*).

**Table 2:** **Genetic map statistics for *E. urophylla *** **and *E. grandis *** **in both the P93 and P97 trials**

	***E. urophylla***		***E. grandis***	
	**P93**	**P97**	**P93**	**P97**
Number of LGs	11	13	11	11
Total map length (cM)	1.502	1.179	1.270	1.021
Average LG length (cM)	137	91	115	93
Number of framework markers	146	106	118	86
Mean distance between markers	11	15	12	15
Number of common framework markers between P93 and P97	72		57	
Missing data (%)	9.7	6.3	8.1	4.2

*LG* linkage group.

Among the framework markers, 72 markers for the female map and 57 markers for the male map were common between P93 and P97 maps. These “bridge markers” enabled the identification 13 homologous regions for the *E. urophylla* parent covering 73% (P93) and 90% (P97) of total map length. For *E. grandis*, 11 homologous regions were identified, covering of 79% (P93) and 89% (P97) of the total map length. As expected, the order between markers was well conserved between P93 and P97: 89% and 93% of the common markers presented the same order for the *E. urophylla* and the *E. grandis* maps, respectively. The four genetic maps with links between bridge markers revealing homologous and orthologous regions are represented in Additional file 8.

### Genetic architecture of growth traits

#### QTL detection in P93

A total of 19 QTLs were detected for the female parent (*E. urophylla*) and 9 for the male parent (*E. grandis*) at the genome-wide type I error rate of 10% (Additional file 9). 71% of the QTLs remained significant at the 5% genome-wide error rate. These QTLs were associated with 15 and 8 different traits for *E. urophylla* and *E. grandis* respectively; representing 8 and 6 different regions distributed over 5 different linkage groups for each parental map. For the *E. urophylla* parent, the variance explained by all the detected QTLs associated with one trait ranged from 5.5% (Ht39) to 21.2% (Asym_h) with an average of 11.5% (± 3.4). For the male parent, this proportion of variance ranged from 4.2% (Ht39) to 13.2% (Cir39) with an average of 7.6% (± 3.1). QTLs for *E. urophylla* were located on LG1, LG3, LG4, LG6 and LG7 with two hotspots (LG3 at 11-12 cM and LG6 at 22-27 cM) gathering 16% and 47% of the total number of QTLs respectively. QTLs for *E. grandis* were located on LG1, LG2, LG3, LG5 and LG10 with two hotspots (LG5 at 113-116 cM and LG10 at 27 cM) gathering 22% and 33% of the total number of QTLs, respectively. The distribution of QTLs between the two parents was different, of the seven LGs with QTLs only two (LG1 and LG3) were common to both parents. Two specific QTLs were found for the growth curve parameter on the *E. urophylla* map on LG3 (for Asym_h and lrc_c). No QTL for GCP were found on the *E. grandis* map.

#### QTL detection in P97

Unlike P93, fewer QTLs were detected for the female parent (33) than for the male parent (72) at the genome-wide type I error rate of 10%, while 74% of the QTLs remained significant at 5%. These QTLs associated with 29 and 62 different traits, represented 8 and 9 different regions distributed on 6 and 7 different LGs for *E. urophylla* and *E. grandis*, respectively (Additional file 10). For *E. urophylla*, the variance explained by all the detected QTLs associated with one trait ranged from 5.1% (Cir11) to 15.3% (Cir52_62) with an average of 7.6% (± 2.5). This proportion of variance ranged from 5.1% (Cir8_9) to 17% (Ht52) with an average of 10.5% (± 2.9) for the male parent (*E. grandis*). QTLs for *E. urophylla* were located on LG2, LG3, LG5, LG6, LG8 and LG10 with three hotspots (LG5 at 47 cM, LG8 at 41-44 cM and LG8 at 66-68 cM ) gathering 12%, 34% and 16% of the total number of QTLs, respectively. QTLs for *E. grandis* were located on LG2, LG3, LG4, LG5, LG6, LG8 and LG10 with three hotspots (LG2 at 0-10 cM, LG4 at 73 cM and LG8 at 22-42 cM) gathering 6%, 6% and 60% of the total number of QTLs, respectively. All LGs with QTLs were common to both parents except for LG4, for which no QTL was detected in *E. urophylla*. One specific QTL region was found for the GCP located on the *E. grandis* map on LG10 (Asym_c, lrc_c and c0_c). The other GCP QTLs co-located with QTLs for different traits.

**Figure 6:**
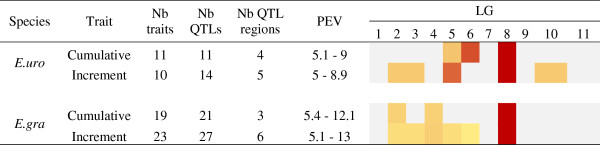
**QTL results for each parental tree of the P97 trial.** The number of QTLs in different genomic regions and their percentage of explained variance (PEV, %) range are given for cumulative growth and growth increment. A colour code indicates the % of QTLs detected in the whole map for each type of trait (from grey = no QTL to dark red = LG with the maximum number of QTLs).

**Figure 7:**
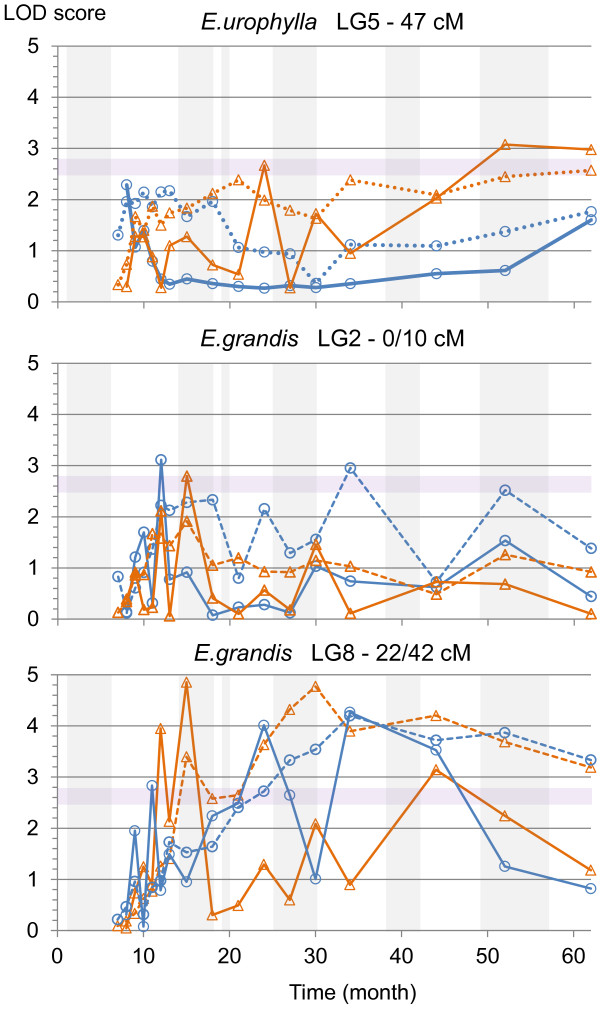
**Evolution of LOD scores at hotspot locations for *E. urophylla *and *E. grandis *in P97.** Cumulative growth is in a dashed line and incremental growth is in a solid line. Height is in blue with circles, circumference is in orange with triangles. The purple zone contains QTLs which are significant at the 10% genome-wide level, above are the QTLs significant at the 5% genome-wide level. Vertical grey bars indicate the dry periods (I_DM_ < 15).

QTLs for growth increment were located on more LGs than QTLs for cumulative growth (Figure 6). The range of phenotypic variance explained by QTLs was similar between cumulative growth and growth increment. For both parents more significant QTLs (5% at genome wide level) were found for growth increments. These genomic regions were located on LG2, LG3 and LG10 for *E. urophylla* and on LG3, LG5 and LG6 for *E. grandis*. In addition to these specific QTLs for growth increments, one QTL region for *E. urophylla* on LG6 was found to be associated with early cumulative growth only (Cir 12, 13, 15, 18). More interestingly, all QTLs for late cumulative growth for both parents co-localized with QTLs for early growth increments, as illustrated for *E. urophylla* on LG5 at 47 cM and for *E. grandis* on LG2 at 0/10 cM and on LG8 at 22/42 cM (Figure 7). Moreover, a co-localization with a QTL for the Asym_c parameter was found for two of these QTLs (*E. urophylla* LG2 and *E. grandis* LG8) suggesting that the genetic architecture of growth response (incremental traits) at the early stage affected the genetic architecture of cumulative growth at the adult stage. Additionally, on LG2, a QTL for the correlation coefficient between growth increment and I_DM_ was found (Position: 0 cM, LOD = 2.55, BCI: 0-21 cM and PEV = 5.34%) for *E. grandis* (P97). This QTL co-located with the hotspot on LG2 for *E. grandis*. For this QTL region, genotypes with a higher growth were also the more responsive (higher Pearson correlation coefficient between Cir_Inc and I_DM_).

#### QTL comparison across trials

**Figure 8:**
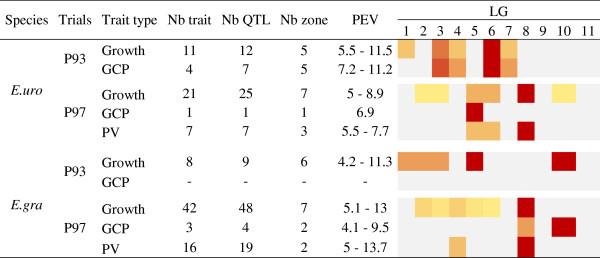
**QTL results for each parental tree in the two trials, P93 and P97.** The number of QTLs in different genomic regions and their percentage of explained variance (PEV, %) range are provided for growth traits (cumulative growth and growth increment), growth curve parameters (GCP) and predicted values (PV) from the monomolecular model. A colour code indicates the % of QTLs detected in the whole map for each type of trait (from grey = no QTL to dark red = LG with the maximum number of QTLs).

Trials P93 and P97 were compared using the growth traits (cumulative and incremental growth), GCPs, as well as specific predicted values (Figure 8). Proportionally to the number of traits, fewer QTL were detected for *E. urophylla* in P97 compared to P93. The proportion of QTLs detected for late growth traits (after 24 months) was higher than for early growth traits for *E. grandis* in both trials and was equivalent for *E. urophylla* in both trials. This may reflect the higher heritability of late growth traits compared with early growth as shown for the same family with P98. Considering P93 and P97, QTLs were detected on all LGs except on LG9 and LG11. However, for LG1 and LG7, QTLs were detected in P93 only. QTLs for GCP were found on three maps (no QTL was detected for *E. grandis* in P93). They were mainly located in growth trait QTL-hotspots except for *E. urophylla* on LG3 and LG8 in P93 and LG10 in P97. QTLs for predicted values were all detected at the same location as other QTLs for growth. Thus, QTLs for predicted values described well the genetic architecture of growth for P97 allowing the comparison with P93.

Co-locating QTLs between P93 and P97 were found on LG2 and 5 for the *E. grandis* parent only, involving strong overlapping of confidence intervals. These two co-localizations involved hotspots of one of two trials but represented few of all the detected QTLs. Indeed a Jaccard Index (I_J_, [86]) was calculated between the two trials (P93 and P97) for *E. grandis* (I_J_ = 12.5%). This difference in the genetic architecture of growth traits between P93 and P97 suggested a strong QTL x environment interaction (QTL x E) that was tested using the predicted values obtained for P97 based on growth curve modelling. Thus, using closest markers already associated with QTLs, a two way analysis of variance was conducted to test QTL x E interaction for growth traits at ages 14, 26, 39, 51 and 59 months for P93 and predicted values at the same ages for P97. GCP were also used for both trials (Additional file 11). We found that most of tested markers revealed a significant interaction with the environment (trial) for both parents, *E. urophylla* 60% and 80%, *E. grandis* 40% and 90% for P93 and P97 respectively. For both trials, QTL x E was more significant for *E. grandis* than for *E. urophylla*.

## Discussion

### Incremental growth at the early stage is related to water availability and determines biomass production at rotation age

We combined the measurement of growth on a sub-annual scale with a monthly characterisation of climate variation using an aridity index (I_DM_), and first showed that seasonal changes in water availability affected secondary and primary growth at the early stage of tree development. Indeed, I_DM_ was found to be strongly correlated with radial growth increment before two years old, while it was less related to growth after two years. Besides, the correlation at early stage was more significant for radial growth (r = 0.69, p < 0.01) than for vertical growth (r = 0.5, p < 0.01), indicating that processes underlying secondary growth are more prone to environmental variation than those involved in primary growth. Other studies have already reported a relationship between apical or radial incremental growth and rainfall on different time scales (day, month and season) in different species [87-89]. In this study, we showed that the first two years corresponded to the highest biomass productivity period, during which 60% of the final height and 65% of final circumference was reached, representing a biomass productivity of 20-27 m^3^.ha^-1^.year^-1^. Therefore, the early growing period constitutes a key period with a strong impact on final growth, as illustrated by the high correlations values between each measurement point and the final growth at 60 months old. In tropical eucalypt plantations, it was found that the maximum growth rate occurred around two years, followed by a decline in production after canopy closure [90-92]. Therefore, our results suggest that climatic conditions underlying the aridity index (mainly rainfall) have a strong impact on early growth response and consequently determine biomass production at rotation age.

### Early growth response to water availability is genetically variable

The small sample size (60 genotypes) used to calculate H^2^ limited the accuracy of the estimation. However, the comparison of time trends in H^2^ between cumulative and incremental growths highlighted contrasting patterns over time which was significant only for Ht. Indeed, cumulative growth displayed an asymptotic increase up to rotation age, contrary to incremental growth which showed high temporal heterogeneity. For *Eucalyptus* grown in Congo, H^2^ for cumulative growth calculated using a wider genetic background were found to range from 0.10 to 0.5 [27] and to stabilize after two or three years. The same trend in H^2^ for cumulative growth was found in *Eucalyptus* in other environments [93-95]. Interestingly, this stabilization of cumulative growth heritabilities occurred at the same time as the decline in wood biomass productivity [92], with a decrease in or stabilization of the G x E interaction [95-97]. It could thus be suggested that the integrative nature of cumulative growth at a mature stage aggregates past genetic responses to environmental variations.

The heterogeneity of H^2^ for incremental growth observed for both height and radial growth suggests a variation in the impact of environmental conditions on growth response. The slowest values of incremental growth heritability were observed during the third dry period. In *Pinus Pinaster*, such heterogeneous trends in H^2^ have been shown for yearly incremental growths [98] and the variability described was significantly linked to environmental effects (year effects). On a lower scale, Parisi [99] also reported a heterogeneous pattern for narrow and broad-sense heritabilities for incremental height growth in *Pinus taeda*. In addition to the contrast between cumulative and incremental growth, H^2^ patterns for growth increment revealed opposite patterns during the 25th and 35th months between primary and secondary growth responses. Costa and Durel [98] also reported a clear difference between primary and secondary growth increments and suggested more stable genetic control for secondary growth response.

Between-tree variation in this early growth response, depicted by the distribution of phenotypic correlations between growth increments and I_DM_ during early growth, suggested a genetic effect as a major component in this response. Other studies have already suggested a genetic effect in response to water availability. Drew *et al*. [17] observed such contrasting behaviour in response to climatic variations (drought) between different *Eucalyptus* hybrids i.e. an *E. grandis* x *E. urophylla* susceptible to drought with intermittent growth, and a less susceptible *E. grandis* x *E. camaldulensis* maintaining a continuous growth. Similar results were found between *E. alba* and *E. urophylla* x *E. grandis* in Congo [18], associated with a significant genotype x environment interaction for traits related to height growth and carbon isotope discrimination (δ^13^C). These genetic differences in environmental susceptibility were also suggested by Bouvet *et al*. [100] comparing different eucalyptus clones. Interestingly, in the present study, we found a QTL for the correlation between I_DM_ and radial growth increment, suggesting that early growth response to water availability is indeed genetically controlled.

### The genetic architecture of final growth is driven by the early growth response to the environment

Given the high macro-synteny and colinearity between eucalyptus genomes, we were able to compare the position of the mapped QTLs to that detected in other studies in different genotypes of the same species or different species. This comparison enabled us to identify two LGs where growth QTLs were frequently detected [39,41-43,52]. As already mentioned by Thumma *et al*. [43], and supported by our result, LG5 was found to contain QTLs for height and radial growth in different *Eucalyptus* species [39,41-43,52] as well as QTLs for foliar chemicals [101]. To a lesser extent, LG8, which was associated with both incremental and cumulative growth in our study, was also associated with growth-QTLs in three other studies [41,43,52], as well as QTLs for rooting ability [102] and QTLs for resistance to biotic stresses [103]. In addition to these two main LGs, and in agreement with the oligogenic model, i.e. few genes with large effects detected as QTLs and many genes with small effects controlled quantitative traits [104], QTLs were found to be distributed across the other nine chromosomes [39,41-44,52].

In this study, a similarity index revealed that most of the growth QTLs were not shared between two experimental trials showing different environmental trajectories (see Figure 1). The statistical power of QTL detection and of estimated effects is directly related to sample size, so called Beavis effect [105]. Therefore QTLs with intermediate or small effects are likely to be not detected with a small sample size (e.g. 100 genotypes). Thus one cannot rule out the fact that QTL instability between P93 and P97 is the result of a rather low sample size: 200 and 190 genotypes for P93 and P97, respectively. This lack of QTL stability could also reveal true biological effect, i.e. a genotypic sensitivity to environmental conditions, as supported by the significant QTL x E interaction found in both parents, and more particularly in *E. grandis*. Interestingly, the two studied species display contrasting adaptability to Congolese conditions with *E. urophylla* better adapted than *E. grandis*[4]. The instability of growth-QTLs between trials also contrasted with the QTL stability found for other traits measured on the same offspring (wood properties and δ13C, data not shown). This difference in sensitivity to environmental variations is generally used to explain the greater PEV and larger number of wood property QTLs in comparison to growth related traits [39,42,106]. It has been found that growth-QTLs often presented a higher level of interaction with the environment [47,48,107] than wood property-QTLs [41,52].

The temporal analysis of QTL detection showed the presence of early stage, intermediate and late QTLs. Among these three patterns of QTL expression, a large proportion of QTLs associated with cumulative growth exhibited early or intermediate patterns: 50% (P93) and 75% (P97) for *E. urophylla* and 75% (P93) and 33% (P97) for *E. grandis*. These three QTL patterns were also reported by Emebiri *et al*. [108] on three-year-old pines. This instability over time is frequently reported in forest tree literature in relation to low heritabilities associated with growth. In *E. globulus*, a study also reported a lack of QTL stability over time for diameter at breast height between two and six years [42]. In the same species, Bundock *et al*. [41] found partial QTL stability for stem diameter with the same location of LOD peaks between the diameter at two and six years old. Along the same lines, Thumma *et al*. [43] also reported relatively low stability for growth QTLs (height and diameter) over time (at two different ages) in one family. In our study, partial stability was found on LG2 (*E. grandis*) and LG5 (*E. urophylla*). Moreover, stable QTLs after two or three years old were found on LG6 (*E. urophylla*), LG8 (*E. grandis*) and LG10 (*E. grandis*). QTL stability over the years has also been reported in gymnosperms [50,109] but their measurements were limited to the young stage (two- to ten-year-old trees) and did not therefore reflect the genetic architecture of the whole growth trajectory.

To our knowledge, our study provides the most comprehensive view of the global instability of growth genetic architecture in a forest tree species thanks to a detailed analysis of the temporal and environmental dynamics of growth over the whole rotation period. Indeed, the fine scale characterization of both growth (multiple measurements) and the environment (aridity index) enabled a precise QTL dissection from early to late growth. In the literature, growth increments are mostly calculated on a yearly basis. Consequently, differences in genetic determinism between cumulative and incremental are less noticeable [109,110]. In our study, the wider variety of growth responses captured by short incremental data was instrumental to characterizing the genetic architecture of growth and depicting its temporal plasticity. More remarkably, thanks to the fine characterization of growth, we found that all QTLs detected at the mature stage for cumulative growth, co-localized with QTLs for early growth increment on both parents (LG5 for *E. urophylla* and LG2, LG8 for *E. grandis*). This result, which goes far beyond a simple age-age correlation analysis, suggests a partly common genetic determinism between early growth response to environmental variation and final growth. The QTL for the correlation with I_DM_ detected on LG2 in *E. grandis*, as well as the pattern of LOD scores for QTLs detected for mature growth on P93 (Additional file 12), corroborate this assumption. To sum up, our study shows that the variability of growth plasticity observed at the young stage partly determined the pattern of cumulative growth genetic architecture at rotation age.

## Conclusion

This study showed differences at phenotypic and genetic levels (phenotypic correlations, broad sense heritability, QTLs) between cumulative and incremental growth, for both primary and secondary growth in a fast growing tree species. Growth increments (especially radial growth) were found to be highly responsive to climatic variations (seasonal changes in water availability) over the two first years, contrary to cumulative growth. However, QTL analysis showed a common genetic basis between early growth increments and final growth. Moreover, strong instability was found in growth-QTLs between the two trials, which was also supported by the analysis of QTL x E. These results suggest that early growth responses (apical and radial growth) to soil water availability partially shape the genetic determinism of late growth and thus growth trajectories. Specific genomic regions were also found for growth increments and growth curve parameters, showing the merits of a combined approach with sequential (increment) and global (curve parameters) growth traits to dissect its genetic determinism. Our results highlight the importance of considering growth as a compound trait for a better understanding of its genetic bases, and suggest to combine selection on early and cumulative growth to improve the ability of trees to produce wood biomass in an optimal way.

## Competing interests

The authors have no competing interests to declare.

## Authors’ contributions

FS: DNA extraction and genotyping. JMB, PV: Field trial management. JB: growth modelling, linkage analysis, QTL mapping. JB, JMG, CP, JMB: manuscript preparation. JMG: Funding and overall supervision. All authors read and approved the final manuscript.

## Supplementary Material

Additional file 1 **Growth trajectories of four individuals (1-4) randomly selected from the full-sib progenies of each trial.** The circumference growth trajectories were fitted by the monomolecular model (purple curve). The mean growth curve for all individuals is represented with a blue line. Click here for file

Additional file 2 **Mean trajectory for cumulative height and circumference for the three trials, P93, P97 and P98.** The thick coloured bars along the x-axis represent dry periods (IDM < 15) for each trial (red for P93, blue for P97 and green for P98). Click here for file

Additional file 3 **Summary of phenotypic data for each trial, P93, P97 and P98.** Traits, number of values, minimum (Min), maximum (Max), mean, standard deviation (SD) and coefficient of phenotypic variation (CVp). Click here for file

Additional file 4 **Evolution over time of the CVp for cumulative and incremental growth for P97 and P98.** The thick grey bars along the x-axis represent dry periods (I_DM_ < 15). Cumulative growth is in dashed lines and incremental growth is in solid lines. Height is in blue with circles, circumference is in orange with triangles. Click here for file

Additional file 5 **Matrix of Pearson correlation coefficients.** For all traits: cumulative growth, growth increment, growth curve parameters and predicted values for P93 (A), P97 (B) and P93 (C). Only significant correlation values (p-value < 0.05) are displayed. Click here for file

Additional file 6 **Phenotypic correlations between final growth and current growth for the three trials.** Cumulative growth is in dashed lines and incremental growth is in solid lines. Height is in blue with circles, circumference is in orange with triangles. Vertical grey bars indicate the dry periods. Click here for file

Additional file 7 **Broad sense heritabilities (H**^**2**^**) with confidence interval (CI) for growth related traits.** Click here for file

Additional file 8 **Genetic maps of *E. urophylla *** **and *E. grandis *** **for P93 and P97.** The LG nomenclature corresponds to the one defined by Brondani *et al*. [81]. Distances between markers are indicated in centiMorgans (cM) by a scale on the left side. Common markers between parental maps for P93 and P97 are in bold. Orthologous regions between the P93 and P97 maps are shown in red (*E. urophylla*) or orange (*E. grandis*) hatching. Common markers between the two parents of P93 are in bold and grey, orthologous regions are represented by a grey bar segment. Accessory markers are positioned near the closest framework marker with the distance in brackets. QTLs are represented by boxes extended by lines representing 95% Bayes credible intervals. If multiple QTLs of the same trait type for different ages co-localize, only one QTL is represented with the largest interval and (*) indicates if one or more QTLs were significant at 5% whole-genome level. Accessory markers with an (*) were distorted (p < 0.01). Traits have been explained in the text. Click here for file

Additional file 9 **QTLs detected in P93 by composite interval mapping in *E. urophylla *** **and *E. grandis.*** The trait, the number of genotype used for analysis (n), the linkage group (LG), the position, the LOD value (* = significant at 5% genome-wide level), the percentage of variance explained by the QTL (PEV, %) and the difference between the two QTL allele effects (D) are indicated. Click here for file

Additional file 10 **QTLs detected in P97 by composite interval mapping in *E. urophylla *** **and *E. grandis.*** The trait, the number of genotype used for analysis (n), the linkage group (LG), the position, the LOD value (* = significant at 5% genome-wide level), the percentage of variance explained by the QTL (PEV, %) and the difference between the two QTL allele effects (D) are indicated. Click here for file

Additional file 11 **Analysis of variance for markers associated with a QTL in P93 (A) or P97 (B).** Similar measurement ages or predicted values were used to analyse the two trials. * p < 0.05, ** p < 0.01, *** p < 0.001. Click here for file

Additional file 12 **Evolution of LOD scores at hotspot locations on *E. urophylla *** **and *E. grandis *** **for P93.** Height is in blue with a circle, circumference is in orange with a triangle. The purple zone contains QTLs which are significant at the 10% genome-wide level, above are the QTLs significant at the 5% genome wide level. Vertical grey bars indicate the dry periods (IDM < 15). Click here for file
