# Supplementary material for: Plasticity of primary and secondary growth dynamics in Eucalyptus hybrids: a quantitative genetics and QTL mapping perspective
Source: BMC Plant Biol. 2013 Aug 26;13:120. doi: 10.1186/1471-2229-13-120 (PMC3870978; doi:10.1186/1471-2229-13-120)
Supplement: Additional file 1 — Growth trajectories of four individuals (1-4) randomly selected from the full-sib progenies of each trial. The circumference growth trajectories were fitted by the monomolecular model (purple curve). The mean growth curve for all individuals is represented with a blue line. [file 1471-2229-13-120-S1.pdf]

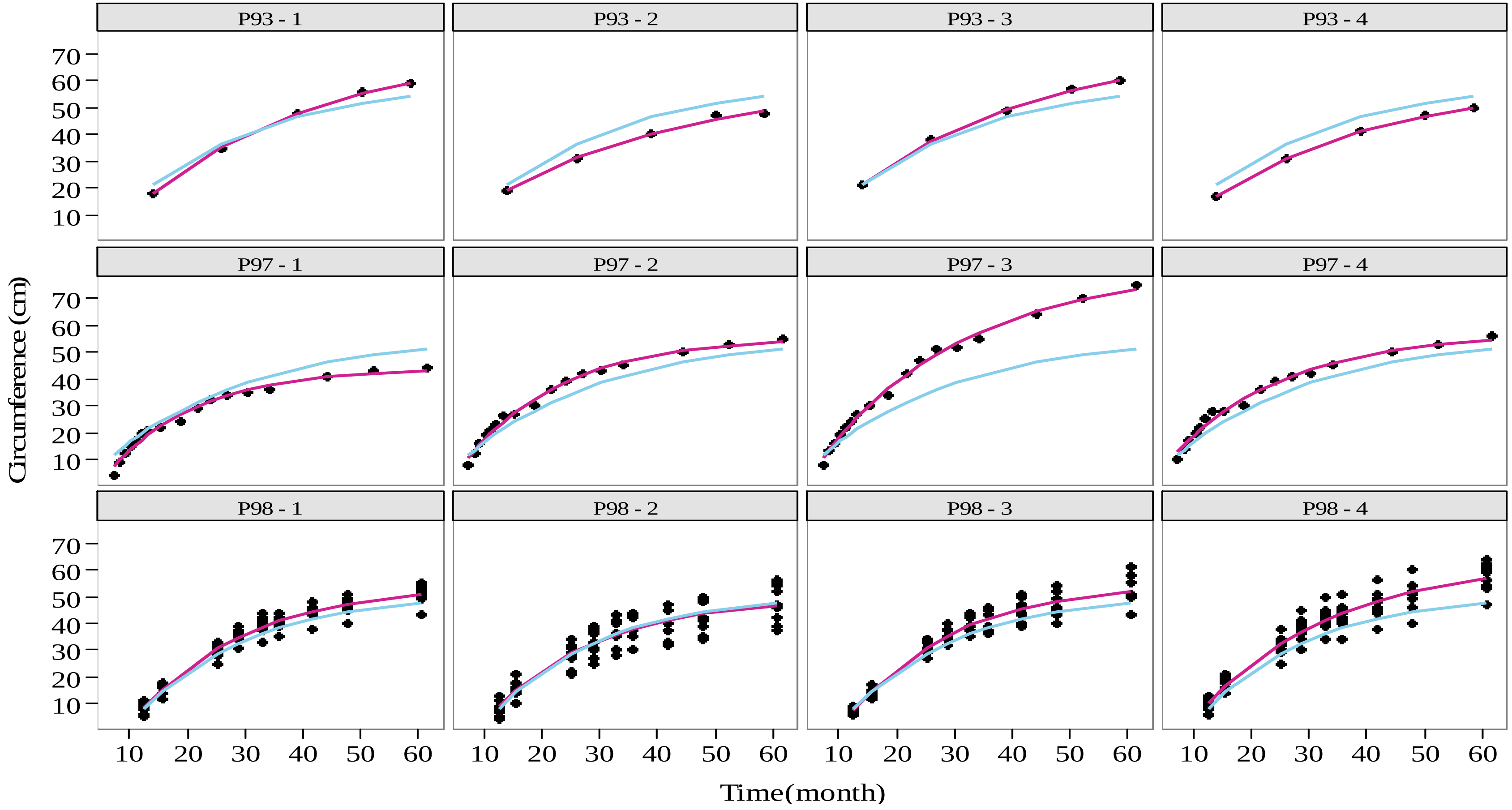

Additional file 1. Growth trajectories of four individuals (1-4) randomly selected from the full-sib progenies of each trial. The circumference growth trajectories were fitted by the monomolecular model (purple curve). The mean growth curve for all individuals is represented with a blue line.
