# Supplementary material for: Plasticity of primary and secondary growth dynamics in Eucalyptus hybrids: a quantitative genetics and QTL mapping perspective
Source: BMC Plant Biol. 2013 Aug 26;13:120. doi: 10.1186/1471-2229-13-120 (PMC3870978; doi:10.1186/1471-2229-13-120)
Supplement: Additional file 2 — Mean trajectory for cumulative height and circumference for the three trials, P93, P97 and P98. The thick coloured bars along the x-axis represent dry periods (IDM < 15) for each trial (red for P93, blue for P97 and green for P98). [file 1471-2229-13-120-S2.pdf]

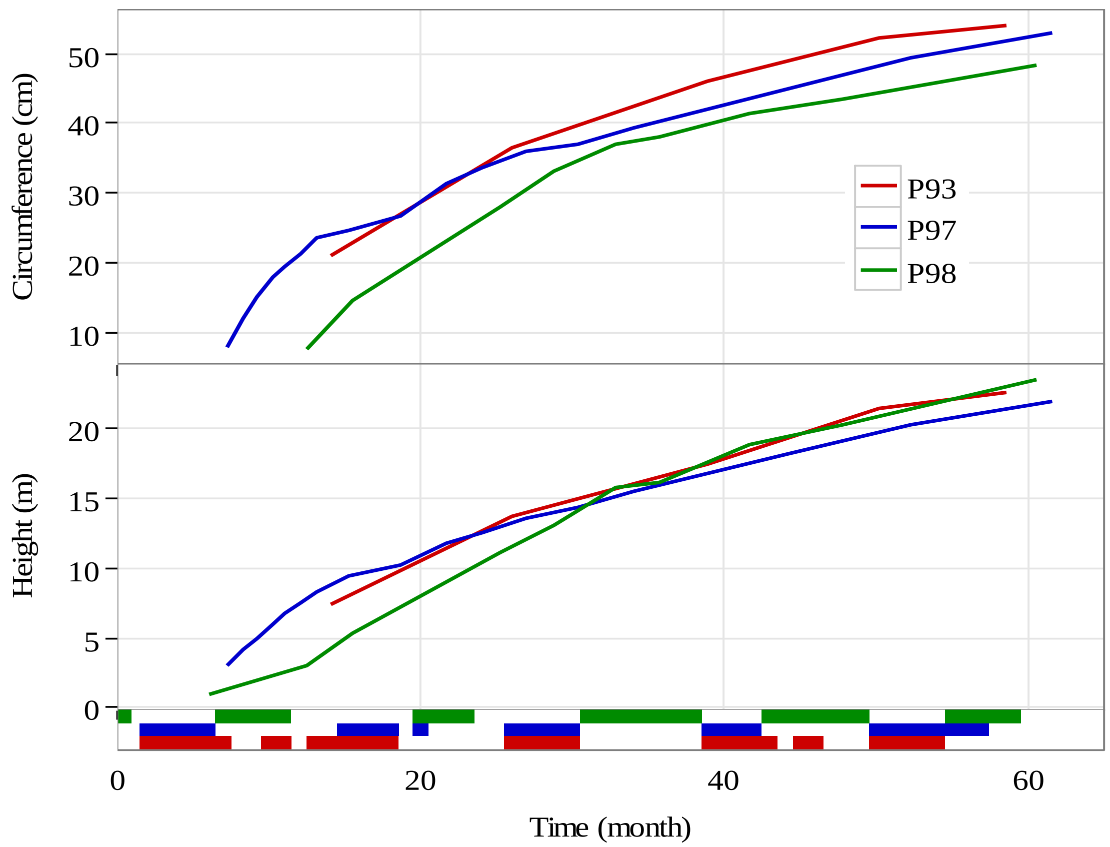

Additional file 2. Mean trajectory for cumulative height and circumference for the three trials, P93, P97 and P98. The thick coloured bars along the x-axis represent dry periods (IDM<15) for each trial (red for P93, blue for P97 and green for P98).
