# Supplementary material for: Plasticity of primary and secondary growth dynamics in Eucalyptus hybrids: a quantitative genetics and QTL mapping perspective
Source: BMC Plant Biol. 2013 Aug 26;13:120. doi: 10.1186/1471-2229-13-120 (PMC3870978; doi:10.1186/1471-2229-13-120)
Supplement: Additional file 3 — Summary of phenotypic data for each trial, P93, P97 and P98. Traits, number of values, minimum (Min), maximum (Max), mean, standard deviation (SD) and coefficient of phenotypic variation (CVp). [file 1471-2229-13-120-S3.pdf]

Additional file 3. Summary of phenotypic data for each trial, P93, P97 and P98. Traits, number of values, minimum (Min), maximum (Max), mean, standard deviation (SD) and coefficient of phenotypic variation (CVp).

HtX =stem heigth at age X months; CirX = stem circumference at 1.30m at age X months; HtX_Y= stem heigth increment between months X and Y; CirX_Y= stem circumference increment between months X and Y; Asym (asymptote of the curve), lrc (logarithm of the time constant) and c0 (value on the abscissa when the curve intersects the x-axis) = 3 parameters of the monomolecular model; P_ = value predicted by the monomolecular model

Trial	Trait	Number of value	Min	Max	Mean	SD	CVp
P93	Ht14	140	5.3	8.8	7.4	0.68	0.09
	Ht26	138	10.3	15.5	13.7	0.93	0.07
	Ht39	198	11.3	20	17.4	1.4	0.08
	Ht51	196	12.5	24.5	21.5	1.72	0.08
	Ht59	198	12.5	28	22.6	2.07	0.09
	Ht14_26	138	0.4	0.7	0.5	0.05	0.09
	Ht26_39	138	0.1	0.4	0.3	0.04	0.15
	Ht39_51	196	0.1	0.5	0.4	0.07	0.19
	Ht51_59	196	0	0.5	0.1	0.07	0.55
	Cir14	141	10	29	21.1	2.98	0.14
	Cir26	139	17	46	36.6	4.58	0.13
	Cir39	198	23	60	46.1	6.6	0.14
	Cir51	198	27	69	52.2	7.83	0.15
	Cir59	197	28	71	54.1	8.26	0.15
	Cir14_26	139	0.6	1.8	1.3	0.2	0.16
	Cir26_39	139	0.2	1.2	0.7	0.17	0.23
	Cir39_51	198	0	0.9	0.6	0.16	0.29
	Cir51_59	197	0	0.6	0.2	0.1	0.49
	Asym_c	138	28.6	88.3	61.7	10.01	0.16
	lrc_c	138	-3.8	-2.2	-3.2	0.24	-0.07
	c0_c	138	-5.6	7.8	3.5	1.86	0.53
	Asym_h	138	11.6	82.6	31.7	6.59	0.21
	lrc_h	138	-5.2	-2.1	-3.7	0.31	-0.08
	c0_h	138	-3.6	8.1	2.6	1.84	0.72
P97	Ht7	189	1	4.4	3.1	0.54	0.18
	Ht8	188	1.1	5.8	4.2	0.64	0.15
	Ht9	187	1.5	6.6	4.9	0.77	0.16
	Ht10	186	2.4	8	6	0.92	0.15
	Ht11	185	2.8	8.8	6.7	1.02	0.15
	Ht12	179	2.9	9.8	7.5	1.16	0.15
	Ht13	171	3.2	10.7	8.3	1.31	0.16
	Ht15	169	3.3	12	9.4	1.64	0.17
	Ht18	167	3.9	13.3	10.2	1.73	0.17
	Ht21	165	4.3	14.3	11.7	1.75	0.15
	Ht24	164	4.8	15	12.5	1.84	0.15
	Ht27	163	6.8	16.5	13.6	1.82	0.13
	Ht30	163	6.8	17.5	14.3	2.06	0.14

Ht34	162	7.8	18.8	15.5	2.22	0.14
Ht44	160	8.5	22	18.2	2.75	0.15
Ht52	157	9	24.5	20.3	3.15	0.16
Ht62	148	10	27.5	21.9	3.51	0.16
Ht13_18	167	0	3.4	1.8	0.69	0.38
Ht24_30	163	0	3	1.7	0.55	0.32
Ht_cum	163	0.1	5.9	3.6	1.06	0.3
Ht7_8	188	0.1	1.5	1.1	0.21	0.18
Ht8_9	187	0	1.2	0.7	0.21	0.29
Ht9_10	186	0	1.6	1.1	0.25	0.22
Ht10_11	185	0	1.7	0.7	0.24	0.33
Ht11_12	179	0	1.3	0.8	0.3	0.37
Ht12_13	171	0	1.4	0.8	0.32	0.41
Ht13_15	169	0	1.2	0.5	0.25	0.48
Ht15_18	167	0	0.6	0.2	0.11	0.45
Ht18_21	165	0	1	0.5	0.19	0.37
Ht21_24	164	0	0.7	0.3	0.13	0.48
Ht24_27	163	0	0.7	0.3	0.13	0.39
Ht27_30	163	0	0.7	0.2	0.14	0.58
Ht30_34	162	0	0.6	0.3	0.12	0.38
Ht34_44	160	0	0.5	0.3	0.09	0.34
Ht44_52	157	0	0.5	0.3	0.09	0.37
Ht52_62	148	0	0.4	0.2	0.08	0.51
Cir7	182	3	14	8.1	2.11	0.26
Cir8	187	3	18	12.2	2.51	0.21
Cir9	186	5	21	15.3	2.68	0.18
Cir10	186	3	23	17.9	3.07	0.17
Cir11	183	5	25	19.6	3.24	0.17
Cir12	179	6	27	21.4	3.55	0.17
Cir13	171	7	30	23.5	3.97	0.17
Cir15	168	7	32	24.7	4.21	0.17
Cir18	165	8	34	26.8	4.49	0.17
Cir21	165	10	42	31.4	5.47	0.17
Cir24	164	11	47	33.6	6.15	0.18
Cir27	163	13	51	36	6.52	0.18
Cir30	163	13	52	37.2	6.86	0.18
Cir34	162	15	55	39.5	7.41	0.19
Cir44	160	16	64	45	9.17	0.2
Cir52	157	18	71	49.3	10.24	0.21
Cir62	151	25	77	52.9	10.78	0.2
Cir13_18	165	0	7	3.1	1.39	0.45
Cir24_30	163	0	6	3.4	1.35	0.4
Cir_cum	162	0	12	6.5	2.42	0.37
Cir7_8	182	0	6	4.3	0.74	0.17
Cir8_9	186	1	5	3	0.78	0.26
Cir9_10	185	0	5	2.7	0.85	0.31
Cir10_11	183	0	3	1.7	0.68	0.41
Cir11_12	178	0	4	1.8	0.79	0.44
Cir12_13	171	0	4	2.1	0.78	0.38
Cir13_15	168	0	1.5	0.5	0.36	0.68

	Cir15_18	165	0	1.7	0.7	0.33	0.49
	Cir18_21	164	0	2.7	1.5	0.55	0.36
	Cir21_24	164	0	1.7	0.8	0.35	0.46
	Cir24_27	163	0	1.7	0.8	0.33	0.43
	Cir27_30	163	0	0.7	0.4	0.21	0.56
	Cir30_34	162	0	1.3	0.6	0.27	0.49
	Cir34_44	160	0	1.2	0.5	0.22	0.42
	Cir44_52	157	0	1.1	0.5	0.22	0.43
	Cir52_62	151	0	0.7	0.3	0.15	0.46
	Asym_c	163	10.6	116.2	55.8	16.59	0.3
	lrc_c	163	-4.9	-0.9	-3	0.57	-0.19
	c0_c	163	-3.8	7	2.1	1.81	0.88
	Asym_h	163	4.9	45.3	25.5	6.54	0.26
	lrc_h	163	-4.8	-1.1	-3.4	0.48	-0.14
	c0_h	163	-6.8	5.9	1.9	1.6	0.84
	P_Ht14	163	3.3	10	8	1.11	0.14
	P_Ht26	163	4.9	16	13.3	1.99	0.15
	P_Ht39	163	5.6	20.9	17.1	2.85	0.17
	P_Ht51	163	5.8	24.3	19.6	3.52	0.18
	P_Ht59	163	5.9	26.1	20.8	3.91	0.19
	P_Ht14_26	163	0.1	0.5	0.4	0.09	0.2
	P_Ht26_39	163	0	0.4	0.3	0.08	0.27
	P_Ht39_51	163	0	0.3	0.2	0.07	0.33
	P_Ht51_59	163	0	0.3	0.2	0.06	0.38
	P_Cir14	163	6.4	27.8	22.6	3.39	0.15
	P_Cir26	163	10.5	47.9	35.2	6.22	0.18
	P_Cir39	163	10.6	61.6	43.2	9.02	0.21
	P_Cir51	163	10.6	69.6	47.6	11	0.23
	P_Cir59	163	10.6	74.3	49.5	12.03	0.24
	P_Cir14_26	163	0.1	1.7	1.1	0.31	0.3
	P_Cir26_39	163	0	1.1	0.6	0.26	0.42
	P_Cir39_51	163	0	0.9	0.4	0.2	0.53
	P_Cir51_59	163	0	0.8	0.2	0.16	0.63
P98	Ht6	546	0.5	1.7	1	0.24	0.24
	Ht12	545	0.7	4.9	3	0.71	0.24
	Ht15	545	1.2	7.7	5.4	1.05	0.2
	Ht25	545	5.1	14.3	11.1	1.48	0.13
	Ht28	545	7.3	17	13.1	1.59	0.12
	Ht33	545	7.8	19.5	15.8	1.94	0.12
	Ht36	544	8.3	20.3	16.2	2.01	0.12
	Ht42	545	8.5	23.8	18.9	2.61	0.14
	Ht48	545	9	25	20.4	2.93	0.14
	Ht60	534	9.5	28	23.5	3.47	0.15
	Ht6_12	545	0	0.6	0.3	0.09	0.27
	Ht12_15	545	0.1	1.1	0.8	0.16	0.2
	Ht15_25	545	0.3	0.7	0.6	0.07	0.12
	Ht25_28	545	0.1	1.1	0.6	0.14	0.22
	Ht28_33	545	0	0.9	0.5	0.13	0.25
	Ht33_36	544	0	0.6	0.2	0.13	0.82
	Ht36_42	544	0	0.9	0.4	0.16	0.36

Ht42_48	545	0	0.6	0.2	0.12	0.49
Ht48_60	534	0	0.5	0.3	0.09	0.37
Cir12	507	3	16	7.7	2.7	0.35
Cir15	543	5	25	14.6	3.86	0.26
Cir25	545	9	41	28.1	5.74	0.2
Cir28	545	11	48	33.2	6.68	0.2
Cir33	545	13	54	37	7.76	0.21
Cir36	544	14	55	38.2	7.96	0.21
Cir42	544	14	62	41.4	9.01	0.22
Cir48	544	15	65	43.5	9.67	0.22
Cir60	534	18	76	48.3	11.1	0.23
Cir12_15	507	0.7	4.3	2.5	0.47	0.19
Cir15_25	543	0.2	2.1	1.4	0.29	0.21
Cir25_28	545	0.3	3.3	1.7	0.45	0.26
Cir28_33	545	0	1.8	0.8	0.29	0.38
Cir33_36	544	0	1.3	0.4	0.26	0.67
Cir36_42	544	0	1.3	0.5	0.25	0.46
Cir42_48	544	0	0.8	0.4	0.17	0.47
Cir48_60	534	0	0.9	0.4	0.18	0.49
Asym_c	545	16.6	87.8	51.4	13.39	0.26
lrc_c	545	-4.2	-2	-2.9	0.26	-0.09
c0_c	545	2.1	13.1	9.5	1.38	0.15
Asym_h	545	9.2	47.6	30.7	6.86	0.22
lrc_h	545	-4.3	-1.6	-3.5	0.32	-0.09
c0_h	545	5.1	21	9.1	1.19	0.13
