# Supplementary material for: Plasticity of primary and secondary growth dynamics in Eucalyptus hybrids: a quantitative genetics and QTL mapping perspective
Source: BMC Plant Biol. 2013 Aug 26;13:120. doi: 10.1186/1471-2229-13-120 (PMC3870978; doi:10.1186/1471-2229-13-120)
Supplement: Additional file 4 — Evolution over time of the CVp for cumulative and incremental growth for P97 and P98. The thick grey bars along the x-axis represent dry periods (IDM < 15). Cumulative growth is in dashed lines and incremental growth is in solid lines. Height is in blue with circles, circumference is in orange with triangles. [file 1471-2229-13-120-S4.pdf]

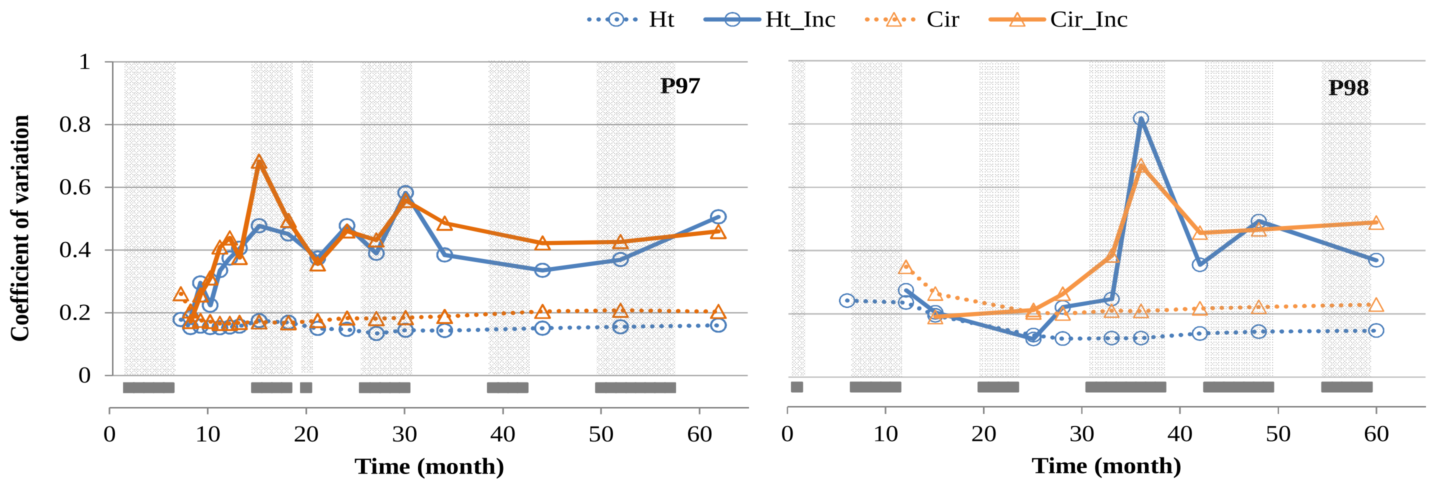

Additional file 4. Evolution over time of the CVp for cumulative and incremental growth for P97 and P98. The thick grey bars along the x-axis represent dry periods (IDM<15). Cumulative growth is in dashed lines and incremental growth is in solid lines. Height is in blue with circles, circumference is in orange with triangles.
