# Supplementary material for: Plasticity of primary and secondary growth dynamics in Eucalyptus hybrids: a quantitative genetics and QTL mapping perspective
Source: BMC Plant Biol. 2013 Aug 26;13:120. doi: 10.1186/1471-2229-13-120 (PMC3870978; doi:10.1186/1471-2229-13-120)
Supplement: Additional file 6 — Phenotypic correlations between final growth and current growth for the three trials. Cumulative growth is in dashed lines and incremental growth is in solid lines. Height is in blue with circles, circumference is in orange with triangles. Vertical grey bars indicate the dry periods. [file 1471-2229-13-120-S6.pdf]

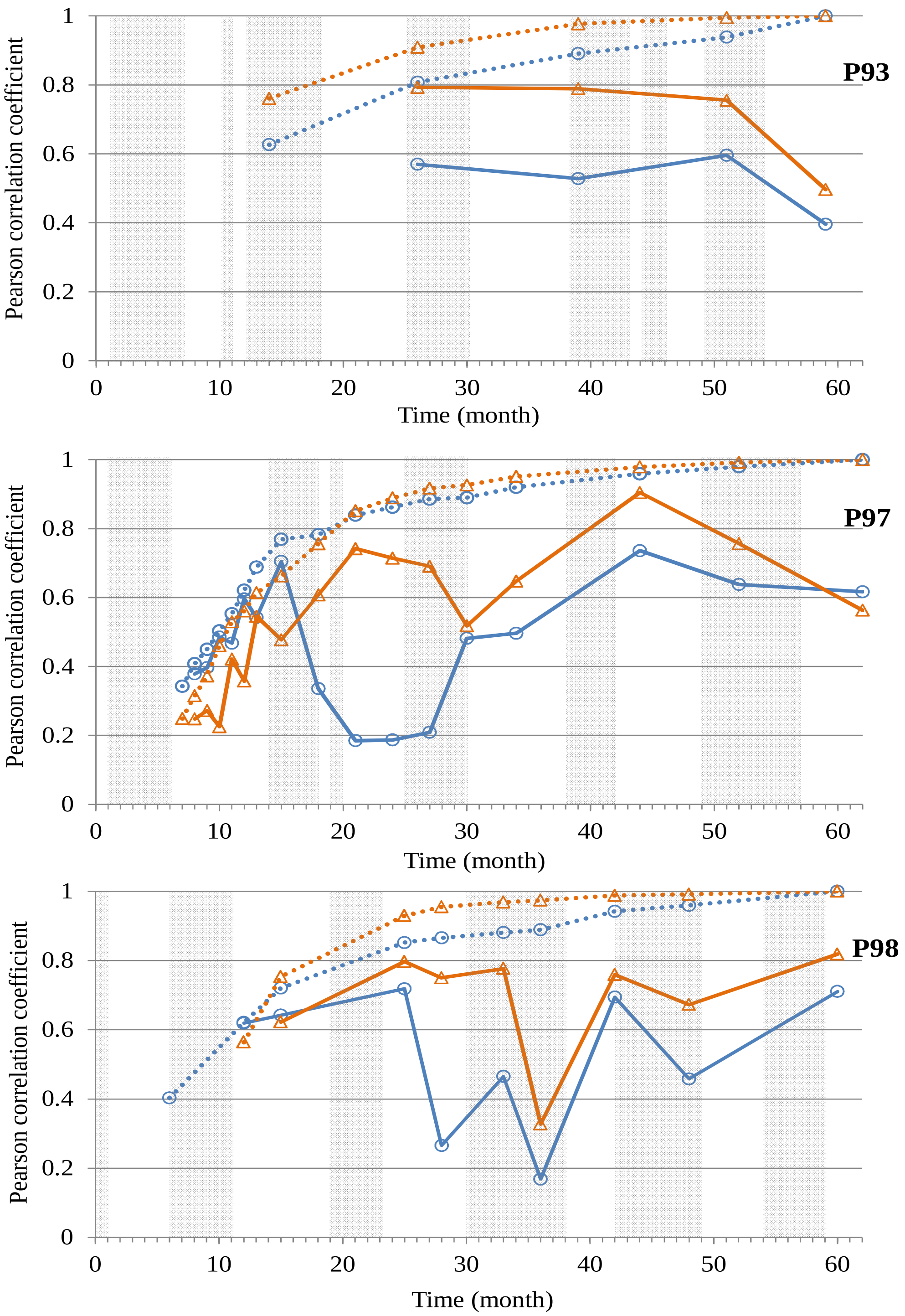

Additional file 6. Phenotypic correlations between final growth and current growth for the three trials. Cumulative growth is in dashed lines and incremental growth is in solid lines. Height is in blue with circles, circumference is in orange with triangles. Vertical grey bars indicate the dry periods
