# Supplementary material for: Plasticity of primary and secondary growth dynamics in Eucalyptus hybrids: a quantitative genetics and QTL mapping perspective
Source: BMC Plant Biol. 2013 Aug 26;13:120. doi: 10.1186/1471-2229-13-120 (PMC3870978; doi:10.1186/1471-2229-13-120)
Supplement: Additional file 7 — Broad sense heritabilities (H 2 ) with confidence interval (CI) for growth related traits. [file 1471-2229-13-120-S7.pdf]

Additional file 7. Broad sense heritabilities (H^2) with confidence interval (CI) for growth related traits.

		Heigth		Height Increment		Circumference		Circumference Increment	
		H^2	CI	H^2	CI	H^2	CI	H^2	CI
Month of measurement	3	0.12	0.04 - 0.19	-	-	-	-	-	-
	6	0.06	0.01 - 0.11	-	-	-	-	-	-
	12	0.16	0.07 - 0.23	0.19	0.08 - 0.25	0.12	0.04 - 0.19	-	-
	15	0.23	0.13 - 0.32	0.26	0.15 - 0.35	0.16	0.08 - 0.24	0.21	0.11 - 0.3
	25	0.29	0.18 - 0.37	0.31	0.2 - 0.39	0.23	0.14 - 0.32	0.26	0.15 - 0.35
	28	0.3	0.17 - 0.37	0.07	0 - 0.11	0.27	0.16 - 0.35	0.34	0.23 - 0.44
	33	0.3	0.19 - 0.39	0.1	0 - 0.16	0.29	0.17 - 0.37	0.29	0.18 - 0.38
	36	0.3	0.19 - 0.39	0	0 - 0	0.29	0.16 - 0.38	0.19	0.1 - 0.27
	42	0.32	0.2 - 0.41	0.28	0.17 - 0.37	0.34	0.22 - 0.42	0.48	0.36 - 0.58
	48	0.35	0.23 - 0.43	0.17	0.08 - 0.25	0.35	0.23 - 0.45	0.27	0.15 - 0.36
	60	0.33	0.2 - 0.41	0.21	0.1 - 0.28	0.38	0.27 - 0.48	0.43	0.31 - 0.52
GCP	Asym	0.35	0.23 - 0.43	-	-	0.44	0.32 - 0.53	-	-
	lrc	0.28	0.17 - 0.37	-	-	0.4	0.28 - 0.48	-	-
	c0	0.14	0.04 - 0.2	-	-	0.14	0.05 - 0.21	-	-
