# Supplementary material for: Plasticity of primary and secondary growth dynamics in Eucalyptus hybrids: a quantitative genetics and QTL mapping perspective
Source: BMC Plant Biol. 2013 Aug 26;13:120. doi: 10.1186/1471-2229-13-120 (PMC3870978; doi:10.1186/1471-2229-13-120)
Supplement: Additional file 8 — Genetic maps of E. urophylla and E. grandis for P93 and P97. The LG nomenclature corresponds to the one defined by Brondani et al. [81]. Distances between markers are indicated in centiMorgans (cM) by a scale on the left side. Common markers between parental maps for P93 and P97 are in bold. Orthologous regions between the P93 and P97 maps are shown in red (E. urophylla) or orange (E. grandis) hatching. Common markers between the two parents of P93 are in bold and grey, orthologous regions are represented by a grey bar segment. Accessory markers are positioned near the closest framework marker with the distance in brackets. QTLs are represented by boxes extended by lines representing 95% Bayes credible intervals. If multiple QTLs of the same trait type for different ages co-localize, only one QTL is represented with the largest interval and (*) indicates if one or more QTLs were significant at 5% whole-genome level. Accessory markers with an (*) were distorted (p < 0.01). Traits have been explained in the text. [file 1471-2229-13-120-S8.pdf]

LG - 1

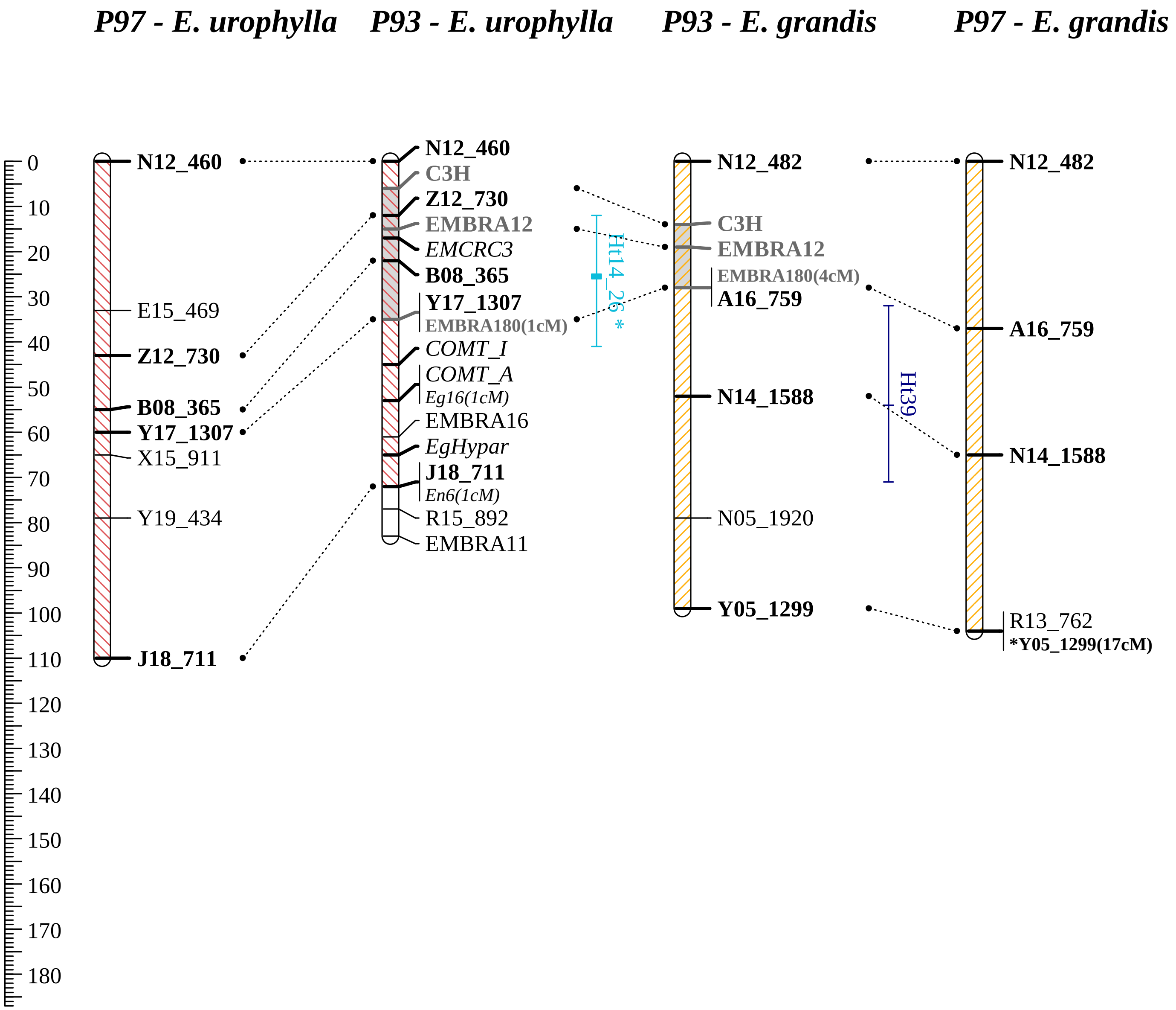

P97 - E. urophylla
P93 - E. urophylla
P93 - E. grandis
P97 - E. grandis
0
10
20
30
40
50
60
70
80
90
100
110
120
130
140
150
160
170
180
N12_460
E15_469
Z12_730
B08_365
Y17_1307
X15_911
Y19_434
J18_711
N12_460
C3H
Z12_730
EMBRA12
EMCRC3
B08_365
Y17_1307
EMBRA180(1cM)
COMT_I
COMT_A
Eg16(1cM)
EMBRA16
EgHypar
J18_711
En6(1cM)
R15_892
EMBRA11
Ht14_26 *
N12_482
C3H
EMBRA12
EMBRA180(4cM)
A16_759
N14_1588
N05_1920
Y05_1299
Ht39
N12_482
A16_759
N14_1588
R13_762
*Y05_1299(17cM)

LG - 2

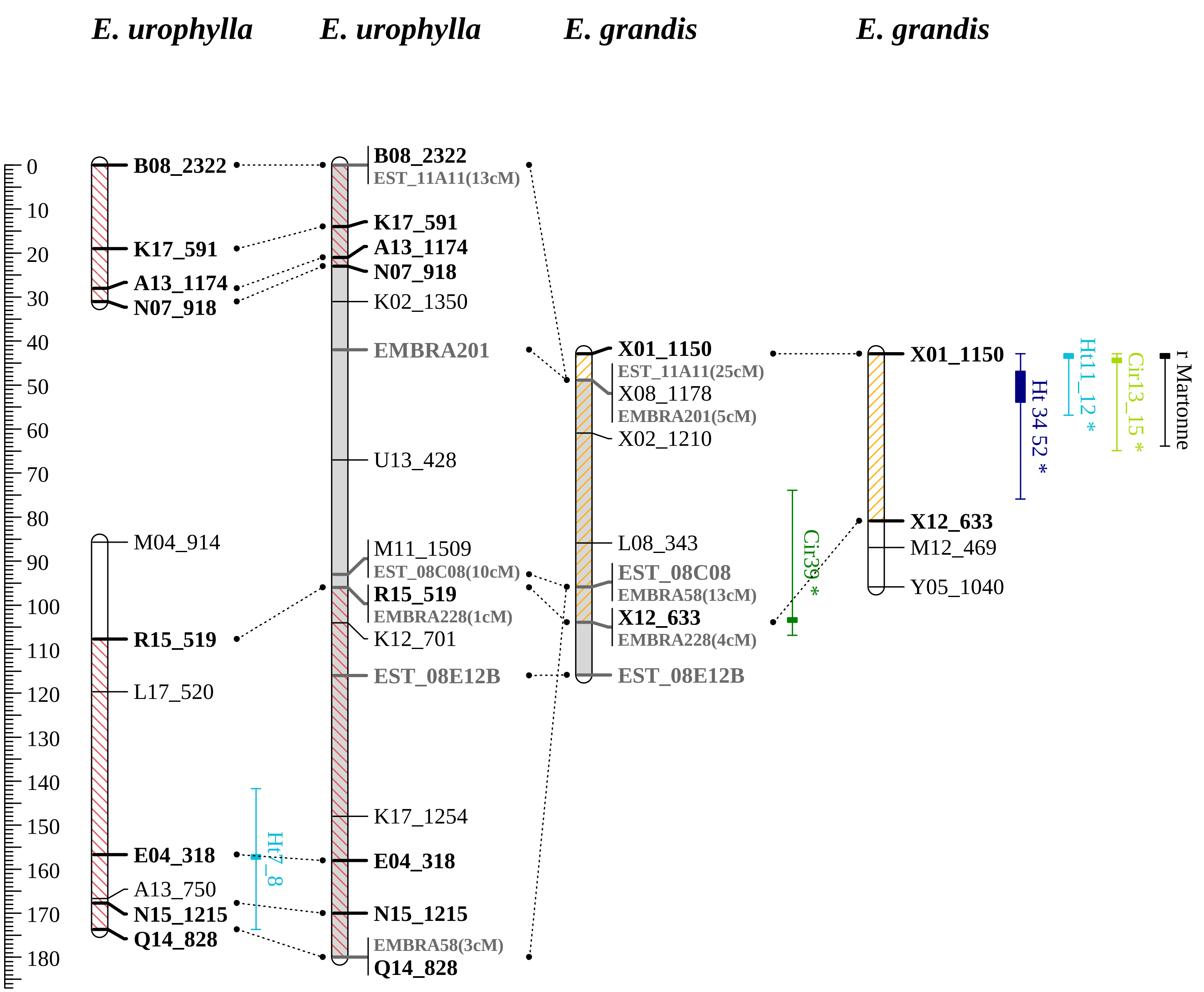

E. urophylla
E. urophylla
E. grandis
E. grandis
0
10
20
30
40
50
60
70
80
90
100
110
120
130
140
150
160
170
180
B08_2322
K17_591
A13_1174
N07_918
M04_914
R15_519
L17_520
E04_318
A13_750
N15_1215
Q14_828
Ht7_8
B08_2322
EST_11A11(13cM)
K17_591
A13_1174
N07_918
K02_1350
EMBRA201
U13_428
M11_1509
EST_08C08(10cM)
R15_519
EMBRA228(1cM)
K12_701
EST_08E12B
K17_1254
E04_318
N15_1215
EMBRA58(3cM)
Q14_828
X01_1150
EST_11A11(25cM)
X08_1178
EMBRA201(5cM)
X02_1210
L08_343
EST_08C08
EMBRA58(13cM)
X12_633
EMBRA228(4cM)
EST_08E12B
Cir39 *
X01_1150
X12_633
M12_469
Y05_1040
Ht 34 52 *
Ht11_12 *
Cir13_15 *
r Martonne

LG - 3

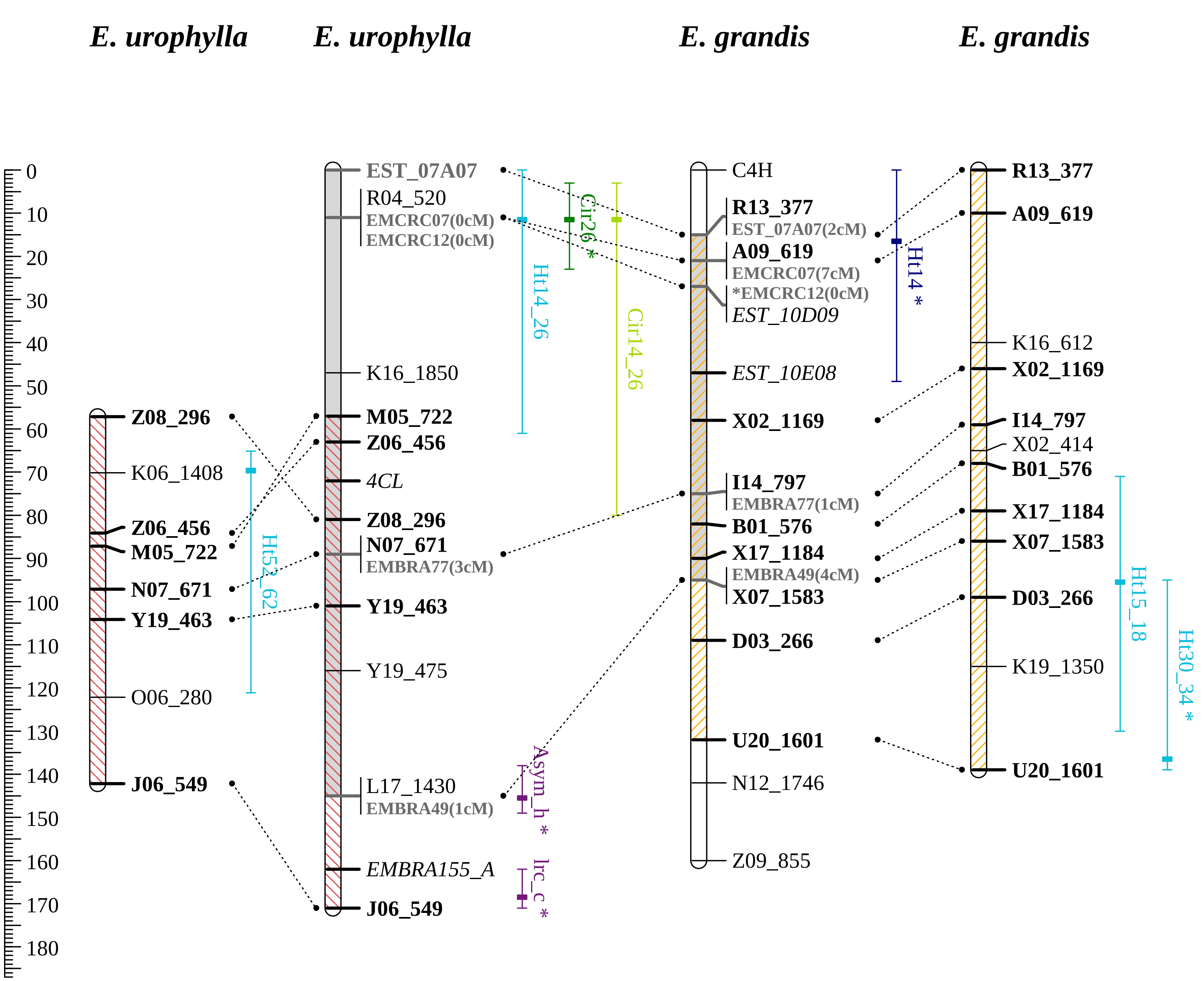

E. urophylla
E. urophylla
E. grandis
E. grandis
0
10
20
30
40
50
60
70
80
90
100
110
120
130
140
150
160
170
180
Z08_296
K06_1408
Z06_456
M05_722
N07_671
Y19_463
O06_280
J06_549
Ht52_62
EST_07A07
R04_520
EMCRC07(0cM)
EMCRC12(0cM)
K16_1850
M05_722
Z06_456
4CL
Z08_296
N07_671
EMBRA77(3cM)
Y19_463
Y19_475
L17_1430
EMBRA49(1cM)
EMBRA155_A
J06_549
Ht14_26
Cir26 *
Cir14_26
Asym_h *
lrc_c *
C4H
R13_377
EST_07A07(2cM)
A09_619
EMCRC07(7cM)
*EMCRC12(0cM)
EST_10D09
EST_10E08
X02_1169
I14_797
EMBRA77(1cM)
B01_576
X17_1184
EMBRA49(4cM)
X07_1583
D03_266
U20_1601
N12_1746
Z09_855
Ht14 *
R13_377
A09_619
K16_612
X02_1169
I14_797
X02_414
B01_576
X17_1184
X07_1583
D03_266
K19_1350
U20_1601
Ht15_18
Ht30_34 *

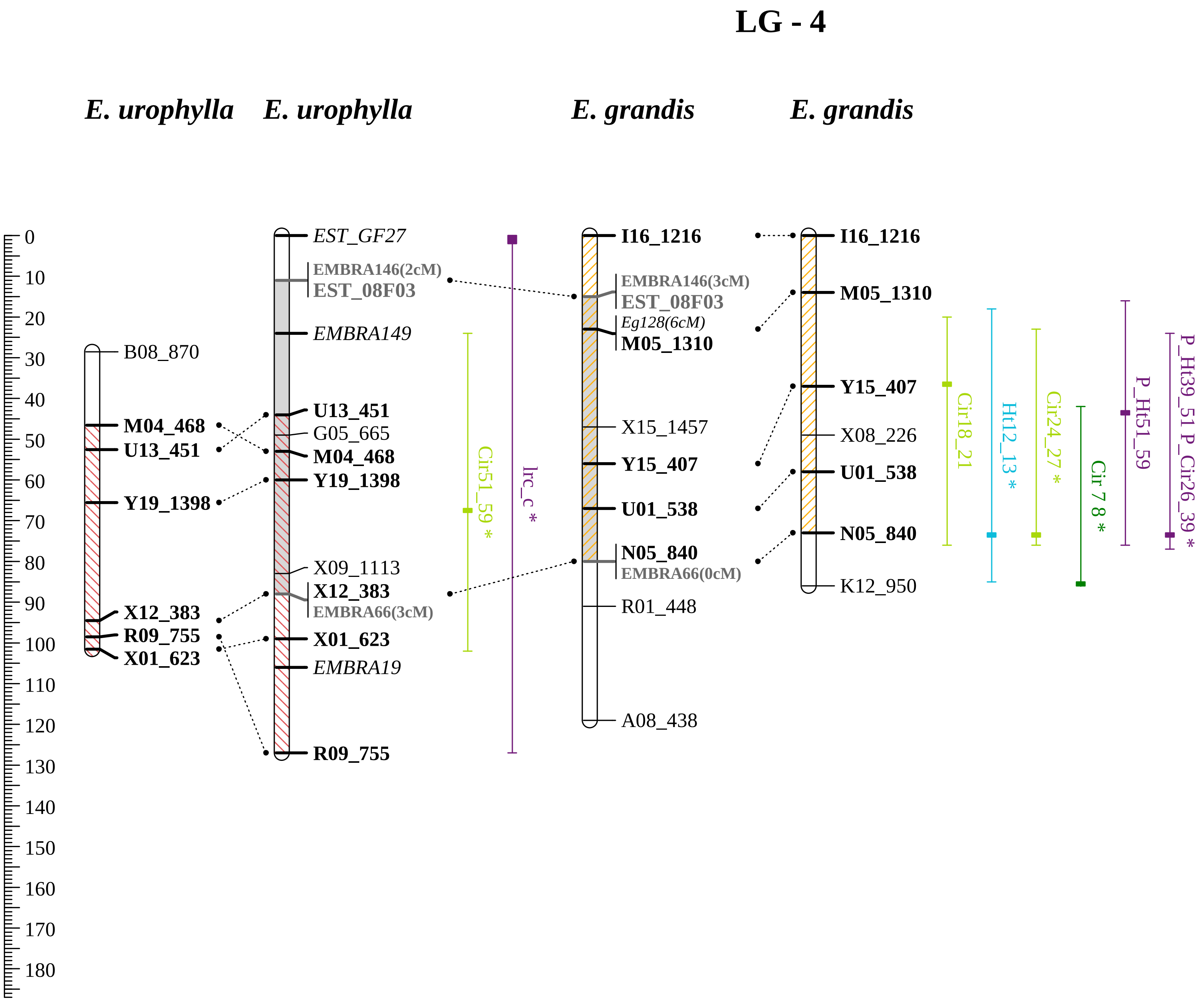
LG - 4
E. urophylla
E. urophylla
E. grandis
E. grandis
0
10
20
30
40
50
60
70
80
90
100
110
120
130
140
150
160
170
180
B08_870
M04_468
U13_451
Y19_1398
X12_383
R09_755
X01_623
EST_GF27
EMBRA146(2cM)
EST_08F03
EMBRA149
U13_451
G05_665
M04_468
Y19_1398
X09_1113
X12_383
EMBRA66(3cM)
X01_623
EMBRA19
R09_755
Cir51_59 *
lrc_c *
I16_1216
EMBRA146(3cM)
EST_08F03
Eg128(6cM)
M05_1310
X15_1457
Y15_407
U01_538
N05_840
EMBRA66(0cM)
R01_448
A08_438
I16_1216
M05_1310
Y15_407
X08_226
U01_538
N05_840
K12_950
Cir18_21
Ht12_13 *
Cir24_27 *
Cir 7 8 *
P_Ht51_59
P_Ht39_51 P_Cir26_39 *

LG - 5

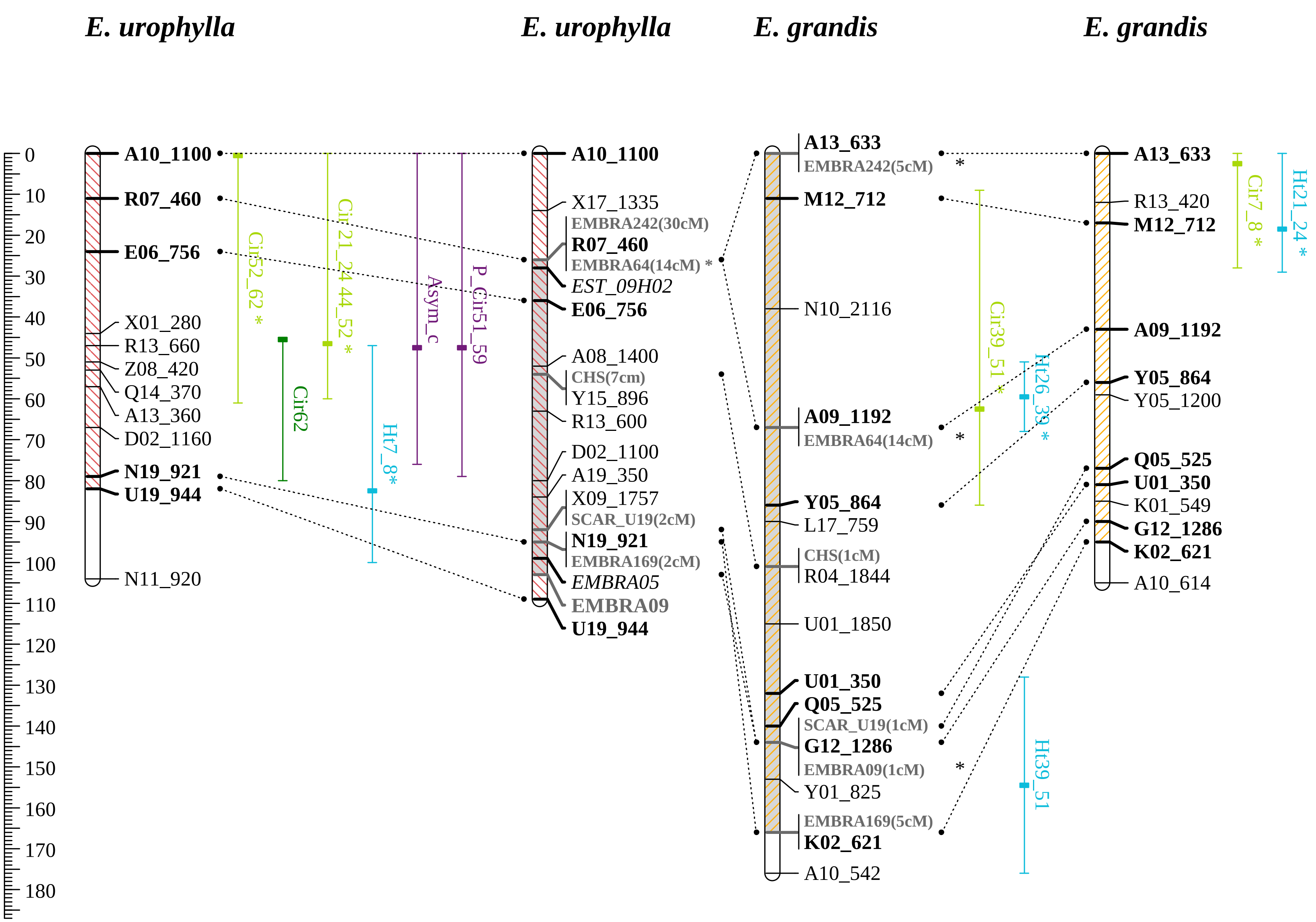
E. urophylla
E. urophylla
E. grandis
E. grandis
A10_1100
R07_460
E06_756
X01_280
R13_660
Z08_420
Q14_370
A13_360
D02_1160
N19_921
U19_944
N11_920
Cir52_62 *
Cir21_24 44_52 *
Cir62
Ht7_8*
Asym_c
P_Cir51_59
X17_1335
EMBRA242(30cM)
EMBRA64(14cM) *
EST_09H02
A08_1400
CHS(7cm)
Y15_896
R13_600
D02_1100
A19_350
X09_1757
SCAR_U19(2cM)
EMBRA169(2cM)
EMBRA05
EMBRA09
A13_633
EMBRA242(5cM)
M12_712
N10_2116
A09_1192
EMBRA64(14cM)
Y05_864
L17_759
CHS(1cM)
R04_1844
U01_1850
U01_350
Q05_525
SCAR_U19(1cM)
G12_1286
EMBRA09(1cM)
Y01_825
EMBRA169(5cM)
K02_621
A10_542
Cir39_51 *
Ht26_39 *
Ht39_51
R13_420
Y05_1200
K01_549
A10_614
Cir7_8 *
Ht21_24 *

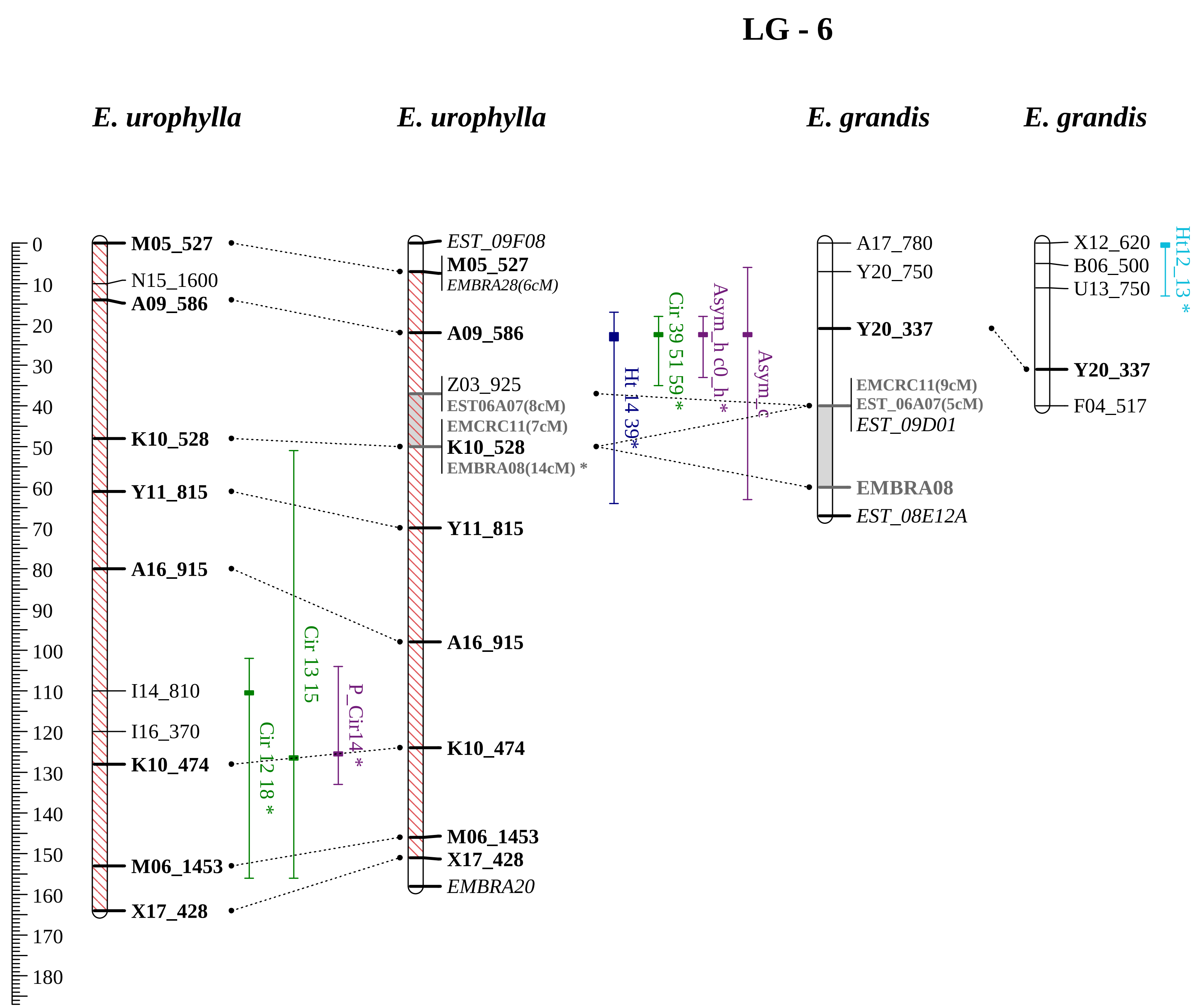
LG - 6
E. urophylla
E. urophylla
E. grandis
E. grandis
0
10
20
30
40
50
60
70
80
90
100
110
120
130
140
150
160
170
180
M05_527
N15_1600
A09_586
K10_528
Y11_815
A16_915
I14_810
I16_370
K10_474
M06_1453
X17_428
EST_09F08
M05_527
EMBRA28(6cM)
A09_586
Z03_925
EST06A07(8cM)
EMCRC11(7cM)
K10_528
EMBRA08(14cM) *
Y11_815
A16_915
K10_474
M06_1453
X17_428
EMBRA20
Cir 13 15
Cir 12 18 *
P_Cir14 *
Ht 14 39*
Cir 39 51 59 *
Asym_h c0_h *
Asym_c
A17_780
Y20_750
Y20_337
EMCRC11(9cM)
EST_06A07(5cM)
EST_09D01
EMBRA08
EST_08E12A
X12_620
B06_500
U13_750
Y20_337
F04_517
Ht12_13 *

LG - 7

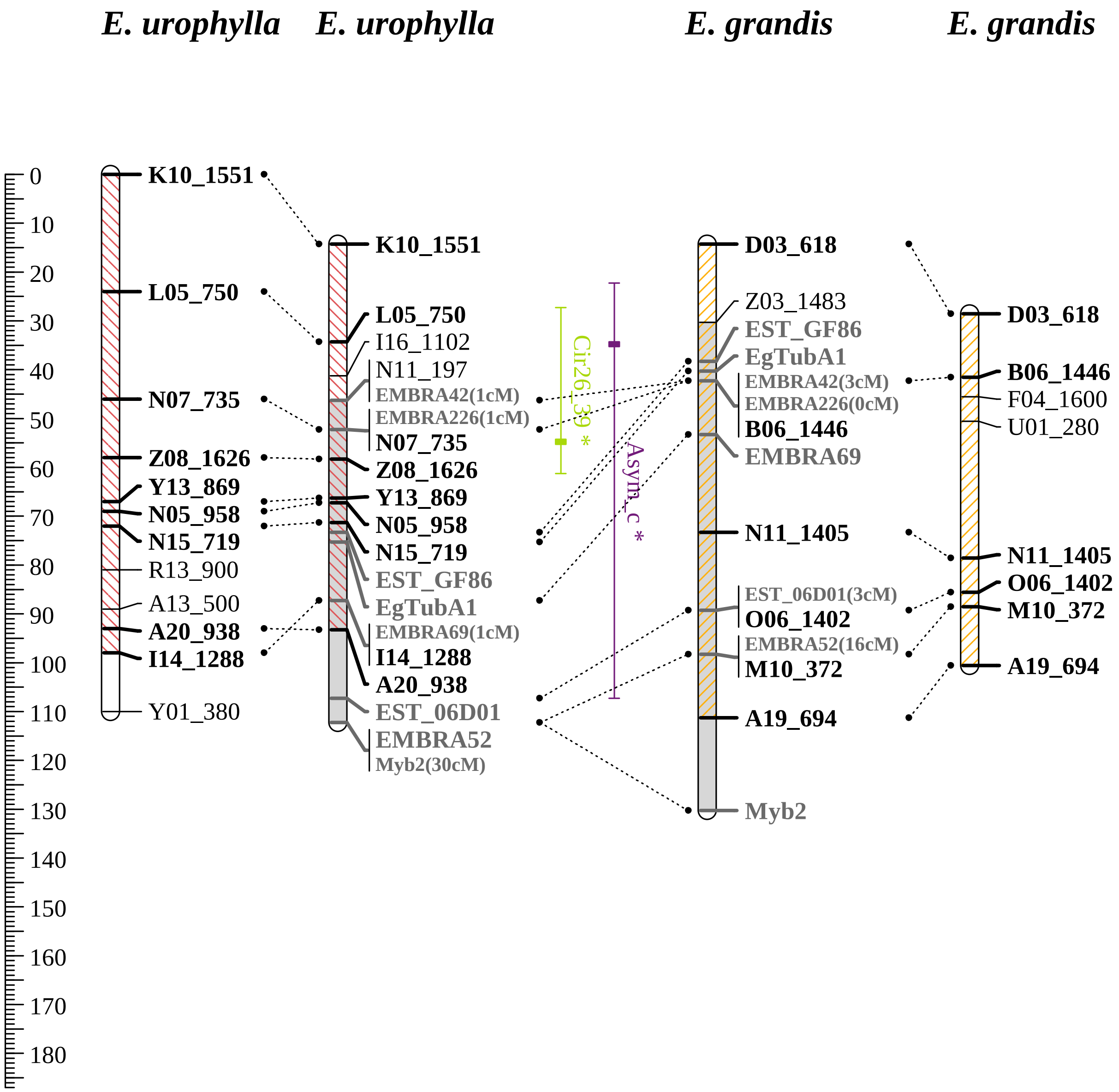

E. urophylla
E. urophylla
E. grandis
E. grandis
0
10
20
30
40
50
60
70
80
90
100
110
120
130
140
150
160
170
180
K10_1551
L05_750
N07_735
Z08_1626
Y13_869
N05_958
N15_719
R13_900
A13_500
A20_938
I14_1288
Y01_380
K10_1551
L05_750
I16_1102
N11_197
EMBRA42(1cM)
EMBRA226(1cM)
N07_735
Z08_1626
Y13_869
N05_958
N15_719
EST_GF86
EgTubA1
EMBRA69(1cM)
I14_1288
A20_938
EST_06D01
EMBRA52
Myb2(30cM)
Cir26_39 *
Asym_c *
D03_618
Z03_1483
EST_GF86
EgTubA1
EMBRA42(3cM)
EMBRA226(0cM)
B06_1446
EMBRA69
N11_1405
EST_06D01(3cM)
O06_1402
EMBRA52(16cM)
M10_372
A19_694
Myb2
D03_618
B06_1446
F04_1600
U01_280
N11_1405
O06_1402
M10_372
A19_694

LG - 8

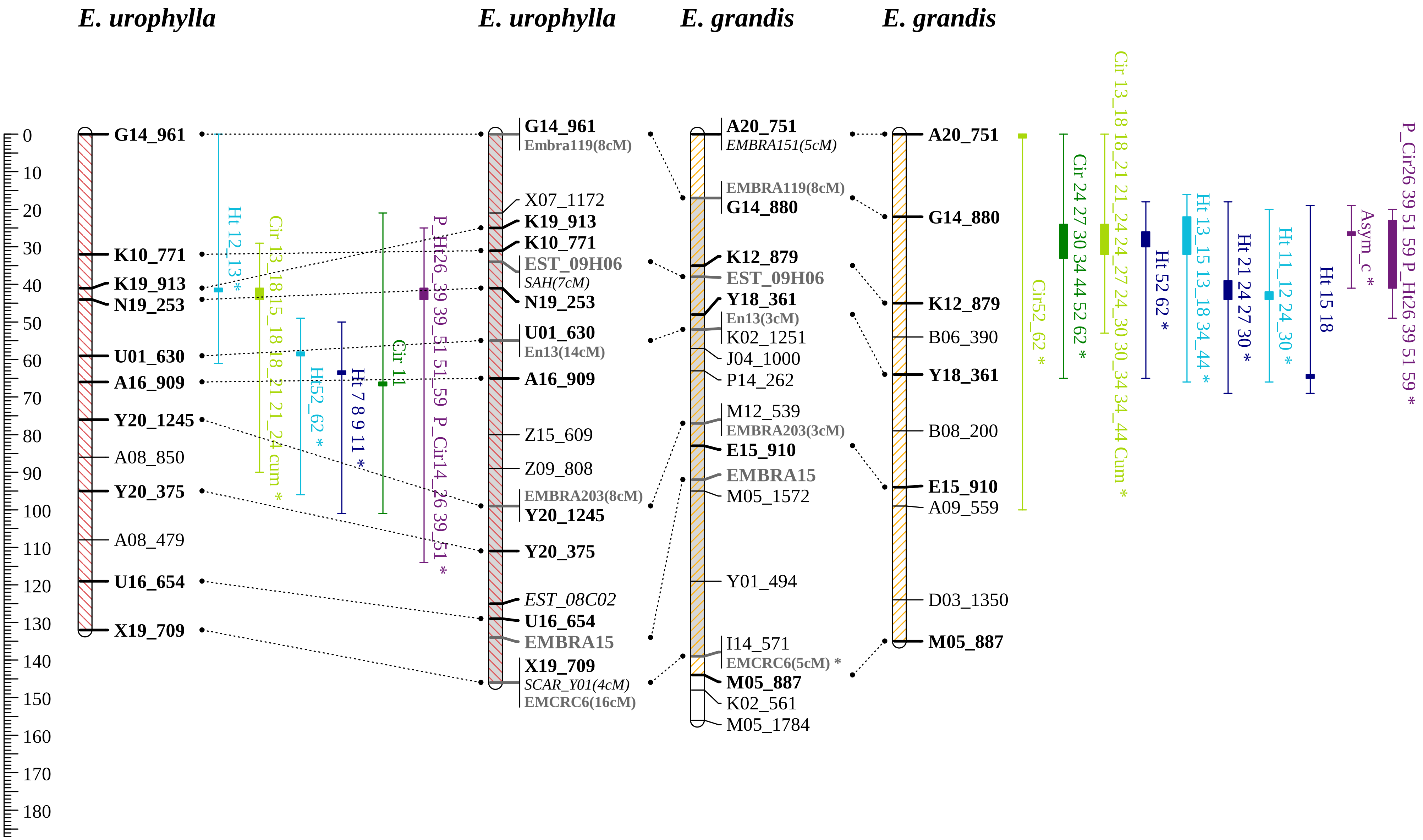

E. urophylla
E. urophylla
E. grandis
E. grandis
0
10
20
30
40
50
60
70
80
90
100
110
120
130
140
150
160
170
180
G14_961
K10_771
K19_913
N19_253
U01_630
A16_909
Y20_1245
A08_850
Y20_375
A08_479
U16_654
X19_709
Ht 12_13 *
Cir 13_18 15_18 18_21 21_24 cum *
Ht52_62 *
Ht 7 8 9 11 *
Cir 11
P_Ht26_39 39_51 51_59 P_Cir14_26 39_51 *
G14_961
Embra119(8cM)
X07_1172
K19_913
K10_771
EST_09H06
SAH(7cM)
N19_253
U01_630
En13(14cM)
A16_909
Z15_609
Z09_808
EMBRA203(8cM)
Y20_1245
Y20_375
EST_08C02
U16_654
EMBRA15
X19_709
SCAR_Y01(4cM)
EMCRC6(16cM)
A20_751
EMBRA151(5cM)
EMBRA119(8cM)
G14_880
K12_879
EST_09H06
Y18_361
En13(3cM)
K02_1251
J04_1000
P14_262
M12_539
EMBRA203(3cM)
E15_910
EMBRA15
M05_1572
Y01_494
I14_571
EMCRC6(5cM) *
M05_887
K02_561
M05_1784
A20_751
G14_880
K12_879
B06_390
Y18_361
B08_200
E15_910
A09_559
D03_1350
M05_887
Cir52_62 *
Cir 24 27 30 34 44 52 62 *
Cir 13_18 18_21 21_24 24_27 24_30 30_34 34_44 Cum *
Ht 52 62 *
Ht 13_15 13_18 34_44 *
Ht 21 24 27 30 *
Ht 11_12 24_30 *
Ht 15 18
Asym_c *
P_Cir26 39 51 59 P_Ht26 39 51 59 *

LG - 9

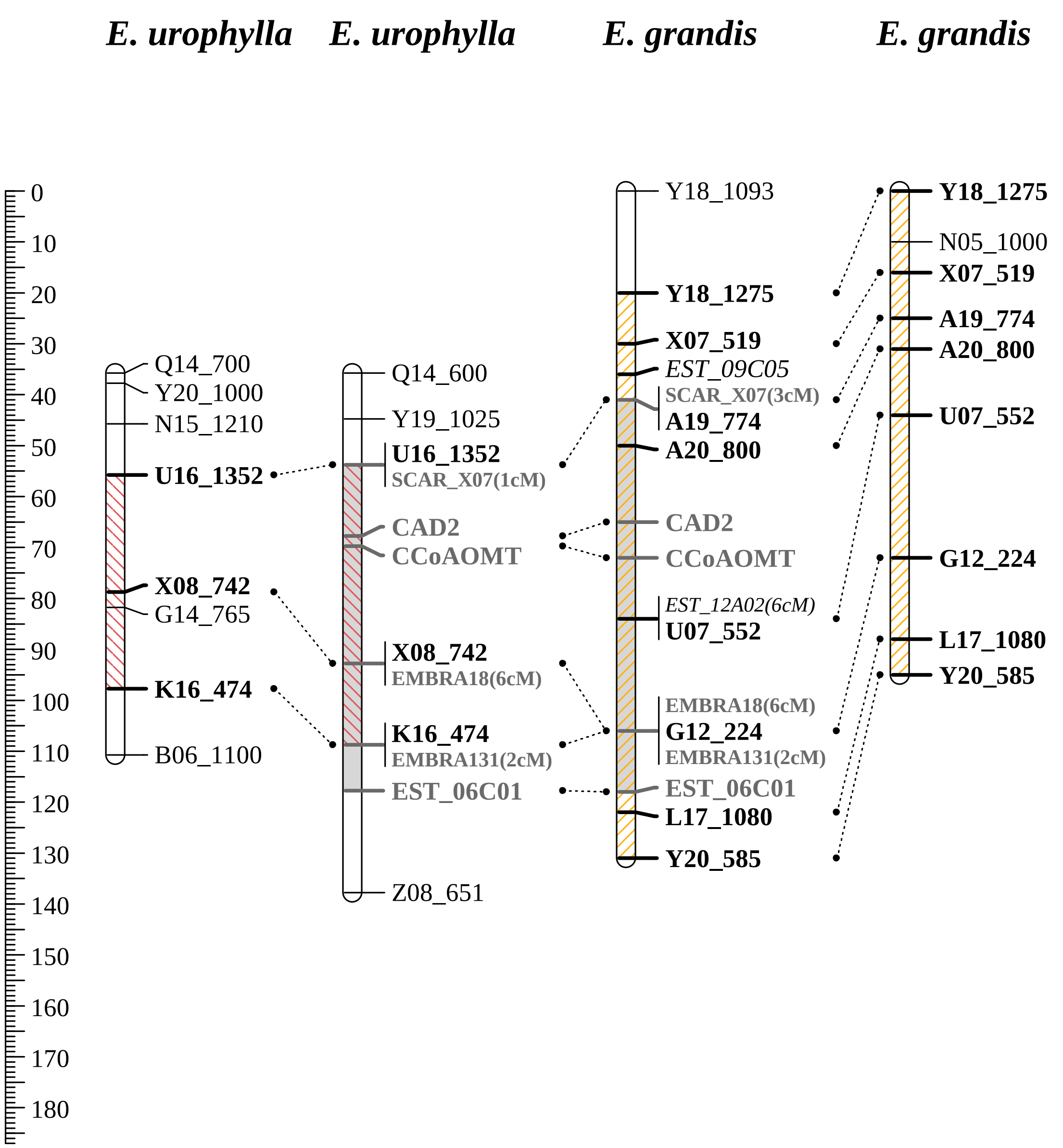

E. urophylla
E. urophylla
E. grandis
E. grandis
0
10
20
30
40
50
60
70
80
90
100
110
120
130
140
150
160
170
180
Q14_700
Y20_1000
N15_1210
U16_1352
X08_742
G14_765
K16_474
B06_1100
Q14_600
Y19_1025
U16_1352
SCAR_X07(1cM)
CAD2
CCoAOMT
X08_742
EMBRA18(6cM)
K16_474
EMBRA131(2cM)
EST_06C01
Z08_651
Y18_1093
Y18_1275
X07_519
EST_09C05
SCAR_X07(3cM)
A19_774
A20_800
CAD2
CCoAOMT
EST_12A02(6cM)
U07_552
EMBRA18(6cM)
G12_224
EMBRA131(2cM)
EST_06C01
L17_1080
Y20_585
Y18_1275
N05_1000
X07_519
A19_774
A20_800
U07_552
G12_224
L17_1080
Y20_585

LG - 10

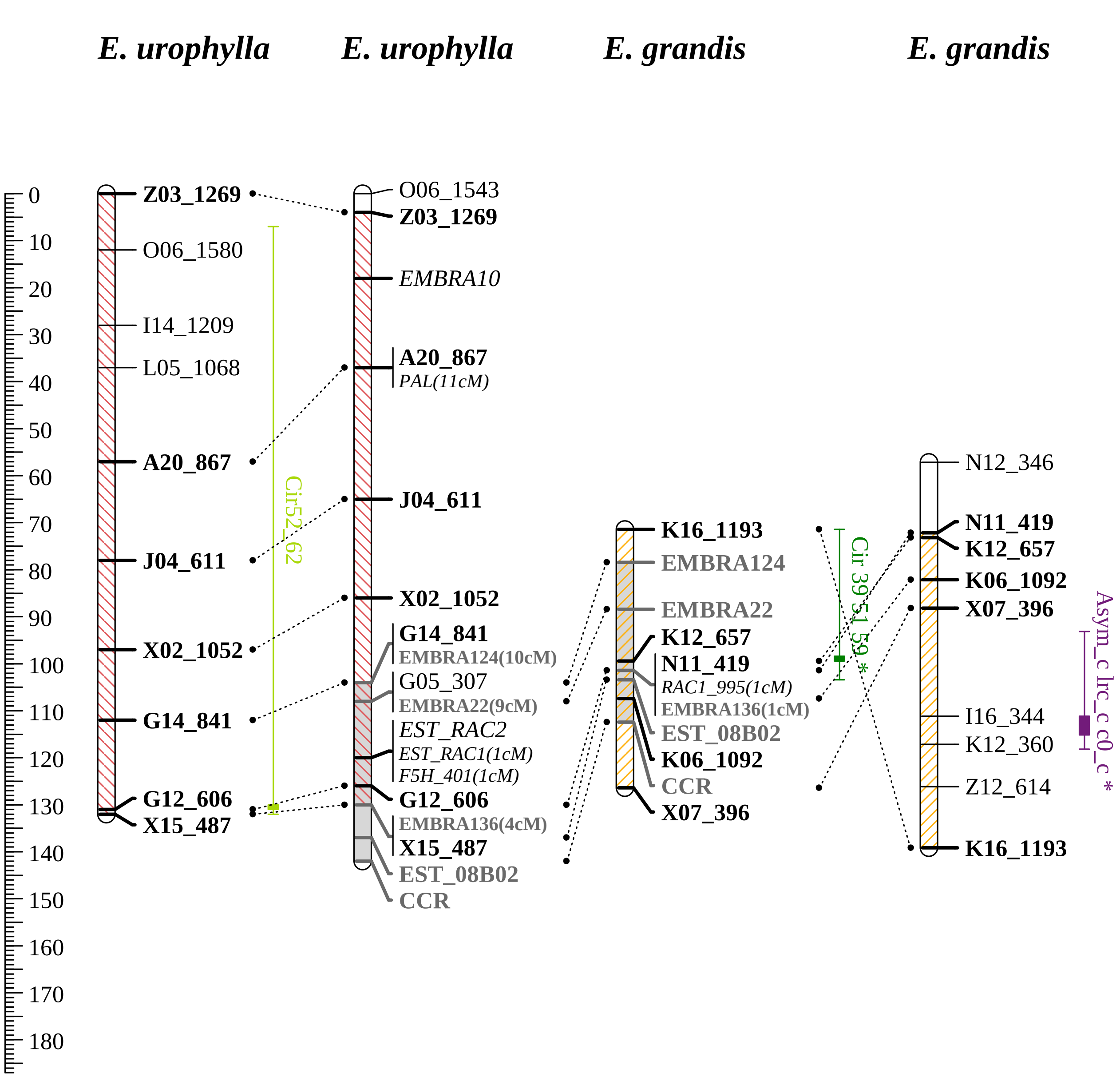

E. urophylla
E. urophylla
E. grandis
E. grandis
0
10
20
30
40
50
60
70
80
90
100
110
120
130
140
150
160
170
180
Z03_1269
O06_1580
I14_1209
L05_1068
A20_867
J04_611
X02_1052
G14_841
G12_606
X15_487
Cir52_62
O06_1543
Z03_1269
EMBRA10
A20_867
PAL(11cM)
J04_611
X02_1052
G14_841
EMBRA124(10cM)
G05_307
EMBRA22(9cM)
EST_RAC2
EST_RAC1(1cM)
F5H_401(1cM)
G12_606
EMBRA136(4cM)
X15_487
EST_08B02
CCR
K16_1193
EMBRA124
EMBRA22
K12_657
N11_419
RAC1_995(1cM)
EMBRA136(1cM)
EST_08B02
K06_1092
CCR
X07_396
Cir 39_51_59 *
N12_346
N11_419
K12_657
K06_1092
X07_396
I16_344
K12_360
Z12_614
K16_1193
Asym_c lrc_c c0_c *

LG - 11

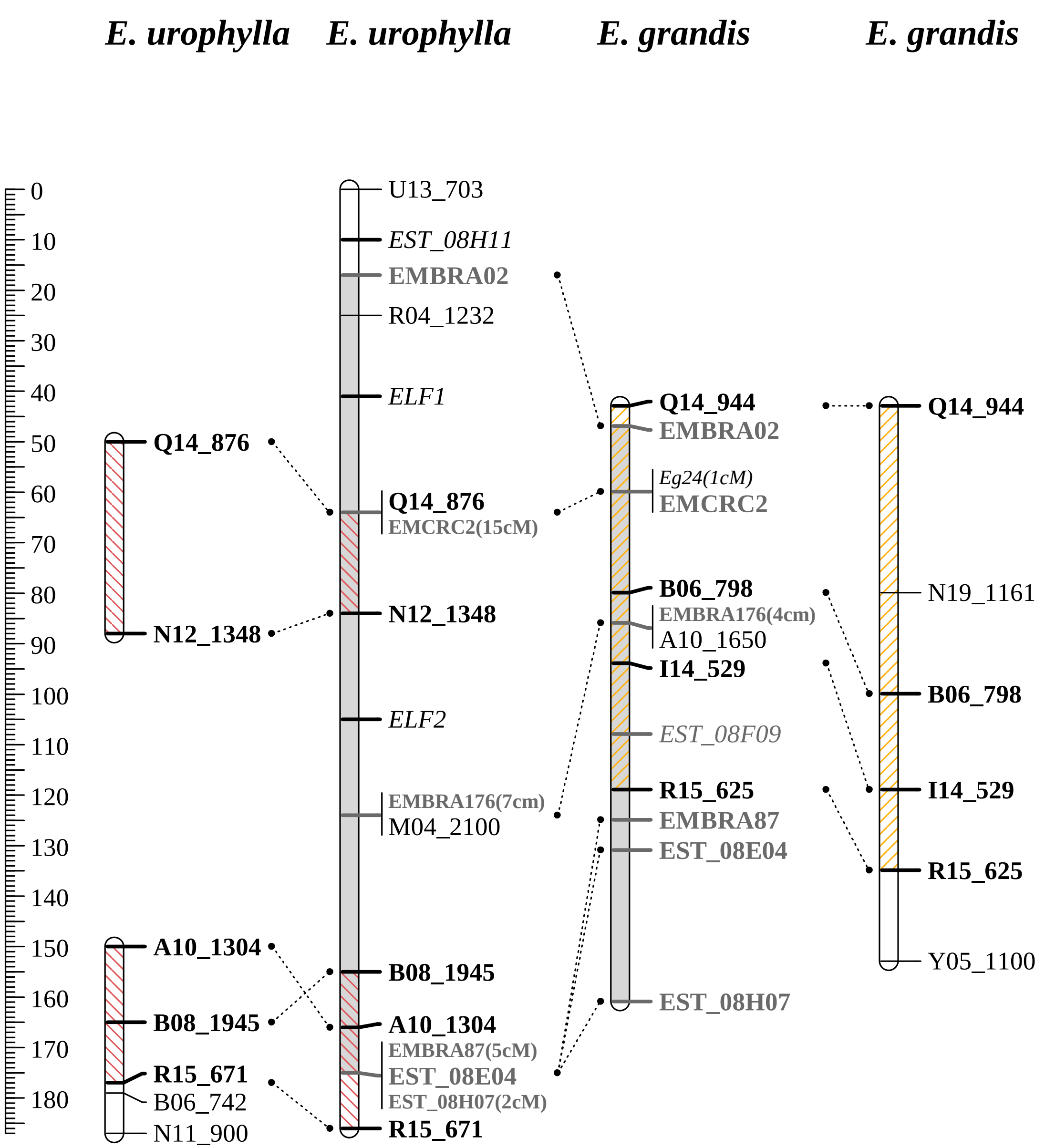
E. urophylla
E. urophylla
E. grandis
E. grandis
0
10
20
30
40
50
60
70
80
90
100
110
120
130
140
150
160
170
180
Q14_876
N12_1348
A10_1304
B08_1945
R15_671
B06_742
N11_900
U13_703
EST_08H11
EMBRA02
R04_1232
ELF1
Q14_876
EMCRC2(15cM)
N12_1348
ELF2
EMBRA176(7cm)
M04_2100
B08_1945
A10_1304
EMBRA87(5cM)
EST_08E04
EST_08H07(2cM)
R15_671
Q14_944
EMBRA02
Eg24(1cM)
EMCRC2
B06_798
EMBRA176(4cm)
A10_1650
I14_529
EST_08F09
R15_625
EMBRA87
EST_08E04
EST_08H07
Q14_944
N19_1161
B06_798
I14_529
R15_625
Y05_1100

Legend

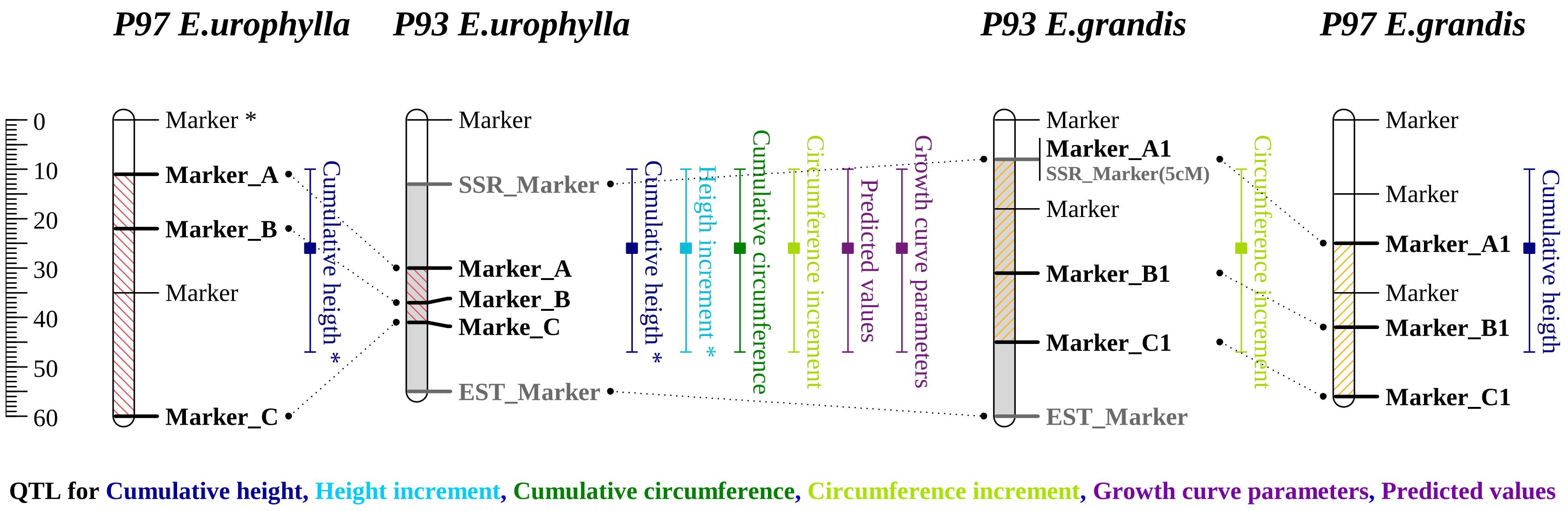

Additional file 8. Genetic maps of *E. urophylla* and *E. grandis* for P93 and P97. The LG nomenclature corresponds to the one defined by Brondani *et al*. [81]. Distances between markers are indicated in centiMorgans (cM) by a scale on the left side. Common markers between parental maps for P93 and P97 are in bold. Orthologous regions between the P93 and P97 maps are shown in red (*E. urophylla*) or orange (*E. grandis*) hatching. Common markers between the two parents of P93 are in bold and grey, orthologous regions are represented by a grey bar segment. Accessory markers are positioned near the closest framework marker with the distance in brackets. QTLs are represented by boxes extended by lines representing 95% Bayes credible intervals. If multiple QTLs of the same trait type for different ages co-localize, only one QTL is represented with the largest interval and (*) indicates if one or more QTLs were significant at 5% whole-genome level. Accessory markers with an (*) were distorted ($p < 0.01$). Traits have been explained in the text
