# Supplementary material for: Plasticity of primary and secondary growth dynamics in Eucalyptus hybrids: a quantitative genetics and QTL mapping perspective
Source: BMC Plant Biol. 2013 Aug 26;13:120. doi: 10.1186/1471-2229-13-120 (PMC3870978; doi:10.1186/1471-2229-13-120)
Supplement: Additional file 9 — QTLs detected in P93 by composite interval mapping in E. urophylla and E. grandis. The trait, the number of genotype used for analysis (n), the linkage group (LG), the position, the LOD value (* = significant at 5% genome-wide level), the percentage of variance explained by the QTL (PEV, %) and the difference between the two QTL allele effects (D) are indicated. [file 1471-2229-13-120-S9.pdf]

Additional file 9. QTLs detected in P93 by composite interval mapping in E. urophylla and E. grandis. The trait, the number of genotype used for analysis (n), the linkage group (LG), the position, the LOD value (* = significant at 5% genome-wide level), the percentage of variance explained by the QTL (PEV,%) and the difference between the two QTL allele effects (D) are indicated

Parent	Trait category	Trait	n	LG	Position (cM)	LOD	BCI	QTL Model	PEV	PEV tot	D
E. uro	Cumulative growth	Ht14	140	6	24	2.904	17 - 64	1	9.47		-0.44
		Ht39	198	6	22	3.161 *	19 - 38	1	5.46		-0.69
		Cir26	139	3	12	3.722 *	3 - 23	1	6.31		-2.27
		Cir39	198	6	22	3.661 *	19 - 35	1	9.65		-4.3
		Cir51	198	6	22	4.857 *	20 - 32	1	10.76		-5.36
		Cir59	197	6	23	4.847 *	20 - 36	1	10.59		-5.61
	Growth Increment	Ht14_26	138	1	26	4.396 *	12 - 41	2	9.47	16.26	-0.34
		Ht14_26	138	3	11	2.797	0 - 61	2	7.79	16.26	-0.3
		Cir14_26	139	3	12	2.917	2 - 80	1	8.38		-1.42
		Cir26_39	139	7	40	3.309 *	13 - 47	1	11.54		1.54
		Cir39_51	198	6	27	4.346 *	21 - 38	1	8.54		-1.08
		Cir51_59	197	4	67	3.096 *	24 - 102	1	7.66		0.53
	Growth Curve	Asym_c	138	6	23	2.963	6 - 63	2	8.36	17.65	-6.7
		Asym_c	138	7	20	3.78 *	8 - 93	2	7.24	17.65	-6.25
		lrc_c	138	3	168	3.557 *	162 - 171	2	7.92	14.02	-0.12
		lrc_c	138	4	0	3.525 *	0 - 127	2	7.28	14.02	-0.12
		Asym_h	138	3	145	3.888 *	138 - 149	2	11.23	21.23	-3.59
		Asym_h	138	6	22	3.272 *	18 - 33	2	8.67	21.23	-3.3
		c0_h	138	6	22	3.218 *	17 - 70	2	8.57	15.18	1.04
E. gra	Cumulative growth	Ht14	140	3	16	3.1 *	0 - 49	1	7.68		0.38
		Ht39	198	1	45	2.63	28 - 67	1	4.17		0.54
		Cir39	198	2	61	3.61 *	31 - 64	2	6.66	13.16	-3.51
		Cir39	198	10	27	3.48 *	21 - 32	2	6.27	13.16	3.39
		Cir51	198	10	27	2.92	0 - 32	1	6.97		4.18
		Cir59	197	10	27	2.76	0 - 32	1	6.88		4.39
	Growth Increment	Ht26_39	138	5	116	4.7 *	108 - 125	1	11.27		-0.39
		Ht39_51	196	5	21	2.77	0 - 48	1	5.62		-0.36
		Cir39_51	198	5	113	3.47 *	90 - 167	1	5.32		-0.82
