# Supplementary material for: Plasticity of primary and secondary growth dynamics in Eucalyptus hybrids: a quantitative genetics and QTL mapping perspective
Source: BMC Plant Biol. 2013 Aug 26;13:120. doi: 10.1186/1471-2229-13-120 (PMC3870978; doi:10.1186/1471-2229-13-120)
Supplement: Additional file 10 — QTLs detected in P97 by composite interval mapping in E. urophylla and E. grandis. The trait, the number of genotype used for analysis (n), the linkage group (LG), the position, the LOD value (* = significant at 5% genome-wide level), the percentage of variance explained by the QTL (PEV, %) and the difference between the two QTL allele effects (D) are indicated. [file 1471-2229-13-120-S10.pdf]

Additional file 10. QTLs detected in P97 by composite interval mapping in E. urophylla and E. grandis. The trait, the number of genotype used for analysis (n), the linkage group (LG), the position, the LOD value (* = significant at 5% genome-wide level), the percentage of variance explained by the QTL (PEV,%) and the difference between the two QTL allele effects (D) are indicated

Parent	Trait category	Trait	n	LG	Position (cM)	LOD	BCI	QTL Model	PEV	PEV tot	D
E.uro	Cumulative growth	Ht7	189	8	66	4.12 *	56 - 96	1	8.55		0.32
		Ht8	188	8	66	3.42 *	56 - 96	1	7.96		0.37
		Ht9	187	8	67	3.49 *	65 - 93	1	7.18		0.42
		Ht10	186	8	86	2.61	49 - 97	1	6.56		0.47
		Ht11	185	8	68	2.72	49 - 97	1	5.93		0.51
		Cir11	183	8	66	2.63	21 - 101	1	5.12		1.49
		Cir12	179	6	110	3.51 *	104 - 132	1	6.02		-1.78
		Cir13	171	6	126	3.49 *	105 - 153	1	6.34		2.02
		Cir15	168	6	126	2.85	51 - 156	1	6.21		2.14
		Cir18	165	6	110	3.02 *	102 - 155	1	6.47		-2.26
		Cir62	148	5	45	2.79	45 - 80	1	9.02		5.93
	Growth Increment	Ht7_8	188	2.2	71	2.58	56 - 88	2	5.03	11.76	-0.1
		Ht7_8	188	5	82	3.28 *	47 - 100	2	6.5	11.76	0.11
		Ht12_13	171	8	41	3.14 *	0 - 61	1	6.52		0.17
		Ht52_62	148	8	58	3.46 *	49 - 96	2	5.8	11.53	0.04
		Ht52_62	148	3	13	2.89	8 - 64	2	5.45	11.53	-0.04
		Cir13_18	165	8	43	3.02 *	39 - 88	1	7.11		0.72
		Cir_cum	162	8	43	3.11 *	39 - 57	1	7.8		1.32
		Cir15_18	165	8	41	2.81	29 - 90	1	6.8		0.17
		Cir18_21	164	8	43	3.52 *	31 - 60	1	8.08		0.31
		Cir21_24	164	8	44	3.42 *	30 - 52	2	8.08	13.77	0.21
		Cir21_24	164	5	47	2.67	0 - 89	2	5.15	13.77	0.17
		Cir44_52	157	5	47	3.08 *	0 - 54	1	5.81		0.11
		Cir52_62	151	10	131	2.87	7 - 132	2	6.28	15.29	-0.07
		Cir52_62	151	5	0	3.07 *	0 - 61	2	8.85	15.29	-0.09
	Growth Curve	Asym_c	163	5	47	2.68	0 - 76	1	6.85		8.54
	Pred	P_Cir14	163	6	125	3.19 *	104 - 133	1	6.45		1.69
		P_Ht26_39	163	8	41	2.83	25 - 114	1	7.18		0.05
		P_Ht39_51	163	8	41	3.32 *	30 - 58	1	7.69		0.04
		P_Ht51_59	163	8	41	3.16 *	26 - 86	1	7.61		0.03
		P_Cir14_26	163	8	44	3.37 *	40 - 55	1	7.05		0.16
		P_Cir39_51	163	8	41	2.7	0 - 88	1	5.5		0.09
		P_Cir51_59	163	5	47	2.7	0 - 79	1	6.22		0.07
E. gra	Cumulative growth	Ht15	169	8	64	3.57 *	24 - 68	1	6.84		-0.85
		Ht18	167	8	64	2.75	19 - 69	1	5.9		-0.83
		Ht21	165	8	39	2.65	17 - 68	1	8.89		-0.98
		Ht24	164	8	44	3.66 *	24 - 66	1	9.44		-1.11
		Ht27	163	8	41	4.32 *	24 - 49	1	11.38		-1.16
		Ht30	163	8	39	4.77 *	23 - 47	1	12.06		-1.33
		Ht34	162	2	11	2.96 *	0 - 33	2	5.81	15.98	-0.98
		Ht34	162	8	43	3.91 *	25 - 49	2	10.37	15.98	-1.38
		Ht44	160	8	22	4.2 *	19 - 41	1	11.08		-1.81

	Ht52	157	2	4	2.52		0 - 33	2	5.43	16.97	-1.63
	Ht52	157	8	26	3.69	*	19 - 65	2	10.1	16.97	-2.06
	Ht62	148	8	30	3.19	*	18 - 65	1	10.28		-2.12
	Cir7	182	4	86	3.87	*	46 - 86	1	9.89		-1.33
	Cir8	187	4	86	2.7		42 - 86	1	6.09		-1.25
	Cir24	164	8	33	2.72		17 - 65	1	10.09		-3.59
	Cir27	163	8	30	3.33	*	19 - 45	1	10.07		-3.82
	Cir30	163	8	24	3.54	*	18 - 41	1	9.44		-4.08
	Cir34	162	8	26	4.2	*	19 - 41	1	10.65		-4.6
	Cir44	160	8	24	3.72	*	18 - 38	1	11.25		-6.03
	Cir52	157	8	24	3.87	*	19 - 38	1	10.02		-6.4
	Cir62	151	8	24	3.33	*	0 - 38	1	8.8		-6.32
Growth	Ht13_18	167	8	25	2.83	*	16 - 66	1	8.73		-0.38
Increment	Ht24_30	163	8	44	3.14	*	20 - 66	1	7.9		-0.31
	Ht_cum	163	8	30	4.18	*	19 - 43	1	11.64		-0.66
	Ht11_12	179	2	0	3.25	*	0 - 14	2	5.87	14.22	-0.15
	Ht11_12	179	8	42	3.95	*	28 - 67	2	8.15	14.22	-0.17
	Ht12_13	171	4	73	2.87	*	18 - 86	2	5.83	15.01	-0.17
	Ht12_13	171	6	40	3.54	*	27 - 40	2	7.99	15.01	-0.19
	Ht13_15	169	8	32	4.85	*	19 - 44	1	13.03		-0.17
	Ht15_18	167	3	95	2.47		71 - 130	1	6.67		-0.06
	Ht21_24	164	5	86	3.29	*	76 - 105	1	7.98		-0.07
	Ht30_34	162	3	136	3.6	*	95 - 139	1	8.75		-0.07
	Ht34_44	160	8	22	3.14	*	17 - 38	1	8.68		-0.05
	Cir13_18	165	8	32	3.36	*	18 - 47	1	6.82		-0.69
	Cir24_30	163	8	26	3.37	*	18 - 42	1	8.1		-0.73
	Cir_cum	162	8	26	4.22	*	19 - 39	1	9.37		-1.42
	Cir7_8	182	5	103	3.86	*	77 - 105	1	7.82		-0.42
	Cir8_9	186	8	64	2.5		15 - 69	1	5.09		-0.35
	Cir10_11	183	8	49	3.05	*	27 - 62	1	6.34		-0.34
	Cir13_15	168	2	2	2.8	*	0 - 22	1	6.62		-0.19
	Cir18_21	164	4	36	2.76		20 - 76	2	6.62	14.44	-0.28
	Cir18_21	164	8	27	2.49		0 - 53	2	7.54	14.44	-0.29
	Cir21_24	164	8	24	4.01	*	19 - 38	1	10.75		-0.22
	Cir24_27	163	4	73	2.8	*	23 - 76	2	7.52	14.08	-0.18
	Cir24_27	163	8	26	2.64		17 - 51	2	7.29	14.08	-0.16
	Cir30_34	162	8	26	4.27	*	19 - 35	1	9.98		-0.17
	Cir34_44	160	8	26	3.53	*	0 - 38	1	9.21		-0.13
	Cir52_62	151	8	0	2.98	*	0 - 100	1	6.41		0.08
Growth	Asym_c	163	8	26	3.52	*	19 - 41	2	9.45	14.82	-10.35
Curve	Asym_c	163	10	28	2.8		22 - 46	2	4.05	14.82	-7.27
	lrc_c	163	10	24	3.65	*	21 - 36	1	8.27		0.33
	c0_c	163	10	24	3.88	*	22 - 34	1	8.06		1.09
Pred	P_Ht26	163	8	41	4.21	*	23 - 49	1	10.45		-1.21
	P_Ht39	163	8	39	4.95	*	23 - 47	1	12.47		-1.84
	P_Ht51	163	8	32	4.94	*	20 - 44	1	13.68		-2.31
	P_Ht59	163	8	27	5.19	*	19 - 40	1	12.88		-2.59
	P_Cir26	163	8	33	3.01	*	19 - 48	1	9.34		-3.47
	P_Cir39	163	8	26	4.32	*	20 - 41	1	11.1		-5.75
	P_Cir51	163	8	26	4.66	*	20 - 40	1	11.77		-7.2

	P_Cir59	163	8	24	3.98 *	19 - 39	1	11.49		-7.92
	P_Ht14_26	163	8	33	5.6 *	24 - 47	1	14.18		-0.05
	P_Ht26_39	163	8	32	5.24 *	21 - 44	1	13.15		-0.05
	P_Ht39_51	163	4	73	2.98 *	24 - 77	2	5.89	16.18	-0.03
	P_Ht39_51	163	8	23	5.32 *	19 - 34	2	10.87	16.18	-0.05
	P_Ht51_59	163	4	43	2.78	16 - 76	2	5.89	14.65	-0.03
	P_Ht51_59	163	8	22	3.58 *	19 - 38	2	7.92	14.65	-0.03
	P_Cir14_26	163	8	30	4.34 *	20 - 43	1	11.65		-0.19
	P_Cir26_39	163	4	73	2.82 *	24 - 78	2	4.96	16.79	-0.11
	P_Cir26_39	163	8	24	4.94 *	20 - 36	2	12.36	16.79	-0.17
	P_Cir39_51	163	8	23	3.64 *	0 - 36	1	10.35		-0.12
	P_Cir51_59	163	8	26	3.54 *	0 - 38	1	9.2		-0.09
Correlation I_{DM}	r I_{DM}	171	2	0	2.56	0 - 21	1	5.34		-0.08
