# Supplementary material for: Plasticity of primary and secondary growth dynamics in Eucalyptus hybrids: a quantitative genetics and QTL mapping perspective
Source: BMC Plant Biol. 2013 Aug 26;13:120. doi: 10.1186/1471-2229-13-120 (PMC3870978; doi:10.1186/1471-2229-13-120)
Supplement: Additional file 11 — Analysis of variance for markers associated with a QTL in P93 (A) or P97 (B). Similar measurement ages or predicted values were used to analyse the two trials. * p < 0.05, ** p < 0.01, *** p < 0.001. [file 1471-2229-13-120-S11.pdf]

Additional file 11. Analysis of variance for markers associated with a QTL in P93 (A) or P97 (B). Similar measurement ages or predicted values were used to analyse the two trials. * p < 0.05, ** p < 0.01, *** p < 0.001

A

Trial	Parent	LG	Marker		P93			Trait P97	Trial effect (p-value)	Marker effect (p-value)	Interaction effect (p-value)
			Name	Position (cM)	Trait	QTL Position (cM)	LOD				
		6	A09_586	22	Ht39	22	3.16	P_Ht39	1.1E-01	8.8E-02	1.7E-01
		6	A09_586	22	Cir39	22	3.66	P_Cir39	9.4E-05 ***	8.7E-04 ***	3.4E-02 *
		6	A09_586	22	Cir51	22	4.86	P_Cir51	6.5E-07 ***	6.1E-04 ***	2.3E-02 *
		6	A09_586	22	Cir59	23	4.85	P_Cir59	6.3E-06 ***	9.7E-04 ***	2.8E-02 *
		6	A09_586	22	Ht14	24	2.9	P_Ht14	1.0E-08 ***	9.7E-03 **	2.8E-01
		6	A09_586	22	Cir39_51	27	4.35	P_Cir39_51	7.9E-22 ***	1.4E-03 **	1.2E-02 *
	E. uro	6	A09_586	22	Asym_h	22	3.27	Asym_h	9.1E-15 ***	1.4E-01	8.3E-02
		6	A09_586	22	c0_h	22	3.22	c0_h	6.6E-03 **	7.9E-03 **	5.0E-02 *
		6	A09_586	22	Asym_c	23	2.96	Asym_c	3.2E-04 ***	4.7E-02 *	6.4E-02
		1	B08_365	22	Ht14_26	26	4.4	P_Ht14_26	9.2E-22 ***	4.1E-01	2.4E-02 *
		3	J06_549	171	lrc_c	168	3.56	lrc_c	4.7E-03 **	5.0E-01	4.6E-02 *
		7	N07_735	38	Cir26_39	40	3.31	P_Cir26_39	4.7E-07 ***	7.0E-04 ***	3.7E-01
		4	Y19_1398	60	Cir51_59	67	3.1	P_Cir51_59	6.1E-03 **	2.5E-02 *	5.4E-02
		10	K12_657	28	Cir39	27	3.48	P_Cir39	2.2E-04 ***	1.1E-01	5.6E-03 **
		10	K12_657	28	Cir51	27	2.92	P_Cir51	1.9E-06 ***	1.0E-01	3.8E-03 **
		10	K12_657	28	Cir59	27	2.76	P_Cir59	1.5E-05 ***	1.3E-01	4.4E-03 **
	E. gra	5	M12_712	12	Ht39_51	21	2.77	P_Ht39_51	3.1E-74 ***	7.4E-04 ***	4.1E-01
		1	N14_1588	52	Ht39	45	2.63	P_Ht39	1.1E-01	6.5E-01	2.1E-02 *
		3	R13_377	15	Ht14	16	3.1	P_Ht14	1.6E-09 ***	1.3E-02 *	3.2E-01
		2	X12_633	61	Cir39	61	3.61	P_Cir39	2.4E-04 ***	1.3E-04 ***	7.6E-01

B

Trial	Parent	LG	Marker Name	Marker Position (cM)	P97 Trait	P97 QTL Position (cM)	P97 LOD	Trait P93	Trial effect (p-value)		Marker effect (p-value)		Interaction effect (p-value)	
		5	A10_1100	0	Cir52_62	0	3.07	Cir51_59	2.7E-12	***	4.9E-04	***	1.4E-02	*
		10	G12_606	131	Cir52_62	131	2.87	Cir51_59	1.6E-17	***	9.6E-04	***	3.5E-02	*
		6	K10_474	128	Cir13	126	3.49	Cir14	7.7E-09	***	3.2E-04	***	9.9E-02	
		6	K10_474	128	Cir15	126	2.85	Cir14	1.3E-15	***	3.6E-04	***	8.8E-02	
	E. uro	8	K19_913	41	P_Ht26_39	41	2.83	Ht26_39	4.6E-01		4.9E-04	***	2.2E-02	*
		8	K19_913	41	P_Ht39_51	41	3.32	Ht39_51	3.4E-74	***	3.4E-04	***	1.3E-01	
		8	K19_913	41	P_Ht51_59	41	3.16	Ht51_59	1.9E-03	**	2.7E-02	*	3.6E-02	*
		8	K19_913	41	P_Cir39_51	41	2.7	Cir39_51	2.4E-21	***	1.3E-02	*	3.6E-02	*
		8	N19_253	44	P_Cir14_26	44	3.37	Cir14_26	7.7E-14	***	1.3E-03	**	1.6E-02	*
		8	A20_751	0	Cir52_62	0	2.98	Cir51_59	3.3E-16	***	2.5E-02	*	2.7E-03	**
		8	G14_880	22	Asym_c	26	3.52	Asym_c	1.7E-04	***	4.5E-04	***	8.8E-04	***
		8	G14_880	22	P_Ht51	32	4.94	Ht51	8.7E-13	***	1.0E-05	***	1.8E-04	***
		8	G14_880	22	P_Ht59	27	5.19	Ht59	2.0E-09	***	2.1E-06	***	8.2E-04	***
		8	G14_880	22	P_Cir26	33	3.01	Cir26	3.9E-02	*	8.0E-04	***	1.9E-02	*
		8	G14_880	22	P_Cir39	26	4.32	Cir39	1.2E-04	***	4.8E-04	***	8.9E-04	***
		8	G14_880	22	P_Cir51	26	4.66	Cir51	7.6E-07	***	1.7E-04	***	7.3E-04	***
P97		8	G14_880	22	P_Cir59	24	3.98	Cir59	6.3E-06	***	1.0E-04	***	6.6E-04	***
		8	G14_880	22	P_Ht14_26	33	5.6	Ht14_26	1.6E-24	***	2.4E-05	***	2.3E-03	**
		8	G14_880	22	P_Ht26_39	32	5.24	Ht26_39	6.0E-01		2.3E-05	***	1.5E-03	**
		8	G14_880	22	P_Ht39_51	23	5.32	Ht39_51	1.2E-74	***	3.1E-03	**	3.4E-03	**
		8	G14_880	22	P_Ht51_59	22	3.58	Ht51_59	2.5E-03	**	2.4E-03	**	7.7E-02	
	E. gra	8	G14_880	22	P_Cir14_26	30	4.34	Cir14_26	5.0E-14	***	5.1E-05	***	8.0E-03	**
		8	G14_880	22	P_Cir26_39	24	4.94	Cir26_39	1.9E-07	***	1.7E-03	**	3.6E-05	***
		8	G14_880	22	P_Cir39_51	23	3.64	Cir39_51	3.5E-22	***	1.2E-04	***	8.2E-03	**
		8	G14_880	22	P_Cir51_59	26	3.54	Cir51_59	1.3E-02	*	4.3E-04	***	3.1E-03	**
		10	K06_1092	24	lrc_c	24	3.65	lrc_c	7.7E-04	***	4.7E-03	**	4.7E-03	**
		10	K06_1092	24	c0_c	24	3.88	c0_c	5.6E-08	***	3.4E-02	*	3.4E-02	*
		8	K12_879	45	P_Ht26	41	4.21	Ht26	2.7E-02	*	1.7E-04	***	9.2E-04	***

8	K12_879	45	P_Ht39	39	4.95	Ht39	1.0E-01		1.3E-06	***	1.5E-03	**
4	N05_840	73	P_Ht39_51	73	2.98	Ht39_51	3.9E-75	***	9.6E-01		2.6E-05	***
4	N05_840	73	P_Cir26_39	73	2.82	Cir26_39	2.3E-07	***	6.7E-02		1.1E-02	*
2	X01_1150	0	Ht52	4	2.52	Ht51	2.0E-06	***	5.2E-04	***	1.1E-02	*
10	X07_396	31	Asym_c	28	2.8	Asym_c	4.4E-04	***	2.5E-01		2.8E-01	
8	Y18_361	64	Ht15	64	3.57	Ht14	3.9E-35	***	4.2E-04	***	9.5E-03	**
