# Supplementary material for: Plasticity of primary and secondary growth dynamics in Eucalyptus hybrids: a quantitative genetics and QTL mapping perspective
Source: BMC Plant Biol. 2013 Aug 26;13:120. doi: 10.1186/1471-2229-13-120 (PMC3870978; doi:10.1186/1471-2229-13-120)
Supplement: Additional file 12 — Evolution of LOD scores at hotspot locations on E. urophylla and E. grandis for P93. Height is in blue with a circle, circumference is in orange with a triangle. The purple zone contains QTLs which are significant at the 10% genome-wide level, above are the QTLs significant at the 5% genome wide level. Vertical grey bars indicate the dry periods (IDM < 15). [file 1471-2229-13-120-S12.pdf]

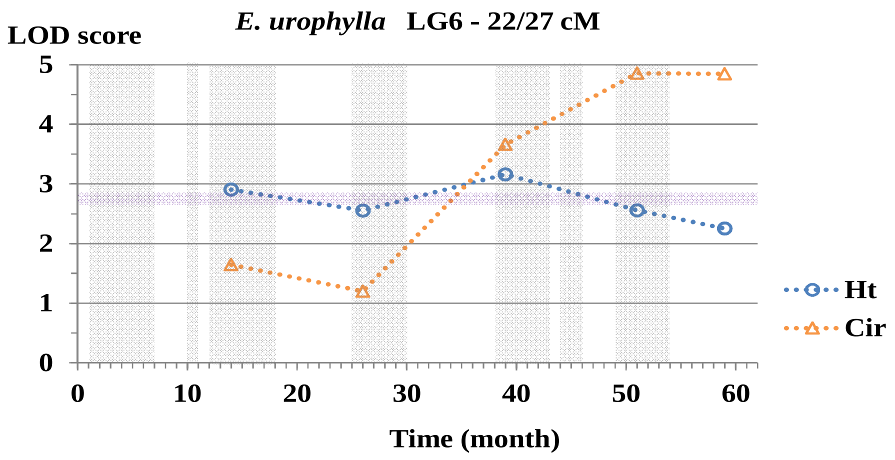

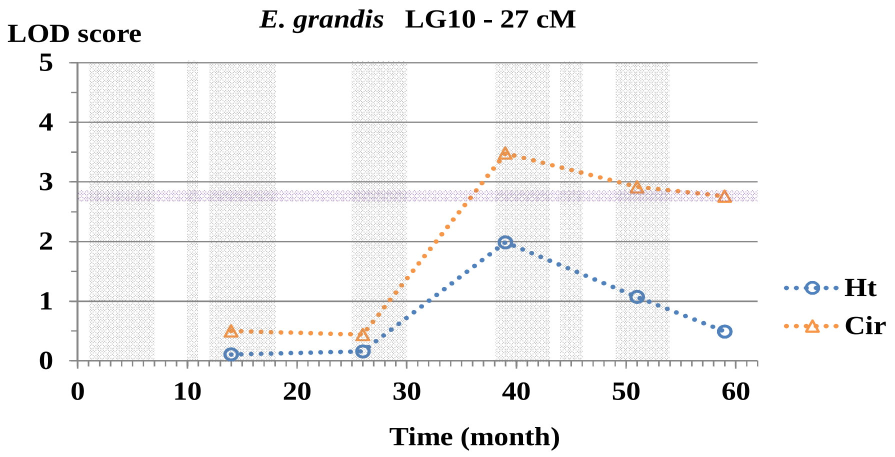

Additional file 12. Evolution of LOD scores at hotspot locations on *E. urophylla* and *E. grandis* for P93. Height is in blue with a circle, circumference is in orange with a triangle. The purple zone contains QTLs which are significant at the 10% genome-wide level, above are the QTLs significant at the 5% genome wide level. Vertical grey bars indicate the dry periods (IDM<15).
